# Delayed infant development: content analysis of a nursing diagnosis proposal^*^

**DOI:** 10.1590/1980-220X-REEUSP-2025-0400en

**Published:** 2026-05-15

**Authors:** Maria do Socorro Távora de Aquino, Samara Pereira Souza Mariano, Flávia Paula Magalhães Monteiro, Huana Carolina Cândido Morais, Vanessa Emille Carvalho de Sousa Freire, Maria Nataniele Queiroz de Lima, Quéren-Hapuque Lopes Sousa

**Affiliations:** 1 Universidade da Integração Internacional da Lusofonia Afro-Brasileira, Instituto Ciências da Saúde, Redenção, CE, Brazil.

**Keywords:** Nursing Diagnosis, Developmental Disabilities, Validation Study

## Abstract

**Objective:**

To analyze the content of the nursing diagnosis “Delayed infant development.”

**Method:**

Methodological study addressing the content analysis phase, using a mixed approach. The first stage was a quantitative agreement study; the second was a qualitative consensus study. Both stages were carried out with proficient individuals. For analysis, the Binomial test was used, with a p value equal to or greater than 0.05 and a cutoff point of 0.85. The level of significance adopted was 5%.

**Results:**

The proposed diagnosis obtained high agreement among the judges regarding its structure (title, definition, defining characteristics, related factors, at-risk populations, and associated conditions), with more than 85% approval. Some characteristics were reformulated to align with NANDA-I, improving clarity and clinical applicability.

**Conclusion:**

The content validity was satisfactory, proving to be relevant, clear, and applicable to nursing practice. Its adoption may favor the early detection of delays in child development and support multidisciplinary interventions, especially in vulnerable contexts.

## INTRODUCTION

Child Development (CD) covers the neuropsychomotor spectrum and can be briefly divided into domains, among which the cognitive, psychosocial, and physical domains stand out, which are interconnected and influence each other throughout an individual’s life^(1)^. Thus, changes in these domains can lead to developmental delays. However, in order for such changes to be detected, the child must undergo continuous monitoring to detect problems early on, or even to monitor the delay once it has already set in, using standardized and validated instruments^(2)^.

Nevertheless, the task of identifying and monitoring children with neuropsychomotor developmental delays faces the complexity of factors such as the lack of an efficient monitoring system, the non-use of standardized and appropriate instruments for screening, or even the cost of tests that are considered the gold standard in the assessment of developmental delays^(3)^.

The period in which these factors will have the strongest influence will also depend on each phase/stage designated according to the child’s life cycle and age. According to the age groups considered by the Ministry of Health, it is during the infant stage, which covers the age from 29 days to 24 months, that the greatest and most rapid changes in CD occur, due to greater brain plasticity. Consequently, children in this stage are also exposed to conditions such as CD delays. However, they also respond better to therapies and stimuli when diagnosed early^(4)^.

Nurses are among the professionals who can screen for CD by using nursing diagnoses to guide their conduct. However, there is no nursing diagnosis that works by specifying each age group and, as listed above, there is a specific age group that is more sensitive to diagnosis and intervention for: the infant stage. Based on this, a previous study^(5)^ constructed the structural elements of a nursing diagnosis focused on developmental delay in infants. However, the content analysis stage, which is essential for the final validation of the diagnosis, had not yet been carried out.

This content analysis refers to one of the stages for constructing or validating a nursing diagnosis, which aims to critically examine its structural elements, such as title, definition, related factors, defining characteristics, at-risk populations, and associated conditions, using psychometric criteria and the participation of expert judges, who ensure that these components are consistent with clinical practice and standardized nursing language, such as that of NANDA-International^(6)^.

It is also worth noting that the use of validated nursing diagnoses allows for the development of more effective care plans, helping to promote child health and prevent diseases. However, for this tool to be applied efficiently, its content must be previously analyzed and evaluated by specialists, ensuring its relevance and clarity in clinical practice.

Against this backdrop, the objective of this study was to analyze the content of the nursing diagnosis “Delayed infant development” using a mixed approach.

## METHOD

### Study Design

Methodological study, addressing the content analysis phase, using a mixed approach with a sequential exploratory design. The first stage was a quantitative agreement study, and the second was a qualitative consensus study. Both stages were carried out with experts in the field. However, the second stage was more rigorous in selecting these judges, considering mainly those with a doctorate who worked/researched in the maternal-child area and/or nursing taxonomies. This was because they would both evaluate the disagreements from the first stage and reevaluate items they deemed relevant.

### Location and Period

The study was conducted remotely. The first stage took place between November 2018 and January 2019, asynchronously, through email contact. The second stage was carried out between October 2020 and January 2021, and took place both asynchronously, also by email, and synchronously, through the Google Meet platform.

### Population, Sample, and Selection Criteria

In both stages, judges/experts were recruited by searching the Lattes Platform of the Lattes Curriculum of researchers, available on the website of the National Council for Scientific and Technological Development (CNPq), using the following terms: concept analysis; nursing diagnoses; child growth and development; child health; content analysis and content validation; and through network or snowball sampling, during which another researcher who could meet the eligibility criteria for this study was requested to be nominated, and the invitation was made via email to each judge.

In order to consider a judge suitable for conducting the evaluation, Fehring^(7)^ criteria were applied in an adapted form, and the recommendations of Lopes et al.^(8)^ were also considered, which mention the balance of participants in terms of clinical experience and theoretical knowledge. Immediately after selection, they were invited and divided by profile: the first group consisted of clinical nurses with at least five years of experience working in child health and child development; and the second group consisted of judges with experience in nursing diagnosis and NANDA International (NANDA-I) taxonomy.

Thus, for agreement analysis (1st stage), the sample size was defined by a calculation that considers the expected proportion of subjects in relation to a dichotomous variable and the maximum acceptable difference in this proportion. The calculation uses the formula: n = Zα2.P.(1-P)/d2, where Zα refers to the confidence level in the interval, adopted at 95%, corresponding to the tabulated value of 1.96; P is the proportion of individuals who agree with the relevance of the nursing diagnosis component, considered to be 85%; and d is the difference in proportion considered acceptable, which will be 15%, including an interval of 70 to 100%. Thus, the final calculation was determined by n = 1.962.0.85.0.15/0.152, resulting in a sample size of 22 judges^(8)^.

At this point, 135 nurses were recruited during the first contact. A total of 36 judges responded, of whom five refused to participate in the study for academic and professional reasons and nine, although they initially accepted, did not return the evaluation within the established deadline and were therefore excluded from the sample. Thus, the final sample consisted of 22 nurse judges.

The second stage of the study was called consensus analysis, as it sought to follow methodological principles characteristic of this type of approach^(9)^. Following the example of the definition of criteria for selecting evaluators, mentioned above, a group discussion strategy was also adopted with the aim of reaching consensus among participants on the diagnostic items. The process involved successive rounds of feedback and collective discussion, in which the judges’ comments and suggestions were considered for the review and improvement of the proposed items, thus characterizing a stage of validation by consensus among experts.

The sampling was non-probabilistic, through the focus group (FG). In this type of approach, the number of judges is determined to be between six and 15. Sixty-one nurses were recruited during the first contact, and 15 judges responded, with eight refusing to participate in the study for academic and professional reasons. The final sample consisted of seven nurse judges.

### Data Collection

After agreeing to participate in the study, each judge received, via email, the Free and Informed Consent Form and the evaluation questionnaire developed in the concept analysis phase, with the elements of the proposed diagnosis “Delayed infant development,” containing the title, definition, defining characteristics, related factors, at-risk populations, and associated conditions. To establish consensus among the judges, with the aim of guiding the indicators to be analyzed in the instrument, relevance was used as one of the psychometric criteria proposed by Pasquali (1999)^(10)^, establishing a Likert scale with five response options, where items 1, 2, or 3 are classified as inappropriate/irrelevant, while items 4 and 5 are considered appropriate/relevant.

In order to establish consensus among the judges collectively, meetings were held via the Google Meet platform, establishing the FG methodology. Two meetings were held, each lasting two hours. The group was led by a mediator, one of the study’s researchers, and both meetings were recorded for later registration and analysis. These same judges had previously evaluated the instrument individually, and the data from the previous evaluation were presented to the FG by the mediator and discussed by both. All questions and suggestions were discussed until consensus was reached, which was considered when the majority of the group agreed with the mediator’s summary of the discussion on each question and suggestion.

### Data Analysis and Processing

The data were compiled in Excel® 2016, and then the Statistical Package for the Social Sciences (SPSS) version 25.0 was used for tabulation, calculation of means, and statistical tests.

The statistical analysis of agreement, according to each item of the instrument, was performed by adjusting the proportions of judges who agreed with the relevance of the instrument. For this purpose, the Binomial test was used, with a p-value equal to or greater than 0.05 with a cutoff point of 0.85. For this analysis, the level of significance (α) adopted was 5%.

### Ethical Aspects

The precepts of Resolution 466/2012 of the Brazilian National Health Council, which deals with research involving human beings, were complied with. The project was registered on the Brazil Platform and approved by CEP/UNILAB, protocol no. 5.032.639.

## RESULTS

For the agreement study, nurses had a 16.2% return rate for the instruments. Of these, 95.5% (n = 21) were female, 59.1% (n = 13) worked in higher education institutions, 63.3% (n = 14) had a doctorate, and 36.4% (n = 08) had a specialization in child health.

Regarding their work in the field of child health research, 77.3% (n = 17) mentioned that they had worked and/or were working in this field, and 45.5% (n = 10) had expertise in the area of nursing diagnosis.

The variables age and length of training showed an asymmetric distribution (p < 0.05), indicating that the median age was 33.50 (±8.78) years, with a minimum age of 27 years and a maximum age of 59 years. Regarding length of training, the median was 10 (±7.21) years, with a minimum of five years and a maximum of 35 years.

In the consensus study, all participants were female. Regarding qualifications, two (28.6%) nurses had specialization in child development, one in neonatology, and all (100%) had doctorates. In addition, the length of training varied between 10 and 20 years. All were working as teachers at universities in various states, such as Ceará, Paraíba, Bahia, Piauí, Goiás, and São Paulo, and worked on topics related to child health and/or nursing taxonomies.

The results of the content evaluation and consensus on the elements of the proposed diagnosis will be organized in tables according to the following topics: evaluation of title and definition, evaluation of the at-risk population and associated conditions, evaluation of defining characteristics, and evaluation of associated factors.

### Title Evaluation and Definition

For content analysis purposes, a diagnosis title option and three definitions previously constructed during concept analysis were sent, along with justification and suggestion options, if deemed necessary. It should be noted that, in the concept analysis stage, the articles selected for the construction of definitions were read thoroughly.

Table 1 shows the judges’ assessment of the appropriateness of the proposed nursing diagnosis title and its respective final definition chosen by the judges.

**Table 1:** Evaluation of the suitability of the title and definition for the proposed nursing diagnosis – Redenção, CE, Brazil, 2022.

Elements	n	%
**Title:** Delayed infant development.	22	100
**Definition:** “Significant delay in two or more areas of development, characterized by alterations that may include motor performance (gross or fine), communication/language, cognitive, social and activities of daily living. Delay is characterized by changes ≥ 2 standard deviations below the mean.”	20	90.9

Table 1 shows the judgment as to the suitability of the title of the proposed nursing diagnosis and its respective final definition chosen by the judges.

It can be observed that there was broad agreement on the two elements presented. However, the consensus analysis experts still suggested some changes to the wording to make it more acceptable to the NANDA-I taxonomy during its submission. Thus, the title became “Delayed Infant Development” and the definition: “Infant presents an absence of two or more milestones in the areas of development, characterized by cognitive, communication/language, and personal/social changes,” justified by being more complete in bringing the domains in which the delay may occur. It is worth noting that all these changes were accepted because they were made by professionals with extensive experience in this area who were evaluating the concept together, and there was consensus among all of them regarding these modifications.

### Assessment of the Adequacy of at-risk Populations and Associated Conditions

Table 2 below presents the indicators that comprised the at-risk populations and associated conditions through concept analysis, according to the judges’ agreement index regarding the relevance criterion.

**Table 2:** Judges’ index of agreement regarding the relevance of indicators for populations at risk and associated conditions – Redenção, CE, Brazil, 2022.

Populations at risk	n	%	p^*^
Social factors (Disadvantaged paternal occupation; Economically disadvantaged family; Lack of access to early childhood education; Children living in developing countries; Little variety of toys; Social isolation of the mother in the first years of the child**’**s life)	22	100	0.028
Unfavorable family environment (Parental stress; Low stimulation; Low maternal schooling; Caregivers without higher education; Presence of 2 or more siblings; Family missing members, especially the mother; First-degree relatives with language delay; Low parent-child interaction)	22	100	0.028
Neonatal conditions (Prematurity; Low weight; Low height; Low head circumference)	22	100	0.028
Obstetric conditions (Age < 20 years; Single mothers; Multiparity; Substance abuse during pregnancy (alcohol, tobacco and other drugs); Unwanted pregnancy; HIV-exposed infants; Prenatal exposure to air pollutants – NO2 and benzene)	21	95	0.137
Genetic factors (Male gender; Non-white ethnicity)	13	59	0.003
**Associated conditions**	**n**	**%**	p^*^
Exposure to drugs (neonatal use of Dexamethasone; prolonged treatment with Hydrocortisone in premature infants; prolonged exposure to anesthetics; exposure to atypical antipsychotics in the prenatal period)	22	100	0.028
Neonatal disorders (5-minute Apgar score <3; Bronchopulmonary dysplasia; Perinatal asphyxia with hypoxic ischemic encephalopathy; Peri-intraventricular hemorrhage; Sequelae of prematurity)	22	100	0.028
Neurological disorders (Infantile epilepsy; Cerebral palsy; Mental retardation)	22	100	0.028
Genetic disorder (Down**’**s disease; Myrhe**’**s disease; Williams**’**disease)	22	100	0.028
Congenital disorders (Congenital heart disease; Congenital profound visual impairment)	22	100	0.028
Head trauma	22	100	0.028
Metabolic disorders	22	100	0.028
Malformation (Cerebral; Craniosynostosis; Cleft lip)	22	100	0.028
Attention Deficit Hyperactivity Disorder (ADHD)	21	95	0.137
Autism	21	95	0.137
Hearing loss	21	95	0.137
Prenatal factors (Placental vein thrombosis; IUGR; Maternal obesity; Gestational diabetes)	21	95	0.137
Infant malnutrition	20	91	0.338
Infections (Cytomegalovirus-infected infants; Otitis; Neonatal sepsis; Neonatal infections)	18	82	0.425
Procedures (Cesarean delivery; Child undergoing cardiac surgery; Admission to neonatal intensive care; Oxygen therapy)	17	77	0.226
Growth delay	17	77	0.028
Vitamin B12 deficiency	17	77	0.226
Eating disorders	17	77	0.226
Maternal disorders (Prenatal and postpartum depression; Postpartum anxiety; Maternal psychological disorder)	16	73	0.000

^*^Binomial *test*.

Considering that the null hypothesis is represented by the agreement between judges equal to 85%, the indicator of populations at risk related to genetic conditions is significant in relation to disagreement between judges and should therefore be removed. Still on this indicator, one judge suggested separating its components (male gender and non-white ethnicity) into different groups, not associating them with genetic conditions.

The other indicators had a concordance index greater than 85% among the judges and a p-value > 0.05 by the binomial test, and thus were approved by the judges. However, there were some suggestions, such as: in “Social factors,” it was suggested to add the elements of environmental conditions, housing, water supply, and the presence of septic tanks and sewers. In “Neonatal conditions,” it was suggested to add high head circumference. In obstetric conditions, the term Sexually Transmitted Infections (STIs) was inserted, in addition to exposure to HIV in infants. In the consensus analysis, the experts maintained their responses. They also emphasized the importance of each element having its associated components. In addition to changing the term “non-white ethnicity” to “black population” within the genetic factors. Therefore, they did not agree on its dismemberment, as previously suggested.

Regarding Associated Conditions, the indicator *child malnutrition* obtained agreement among the judges greater than 85%, and it was suggested that the term “child” be removed. In addition, the indicators *procedures, growth retardation, malformation, vitamin B12 deficiency, eating disorders, and infections* obtained an agreement index of less than 85%. However, for the binomial test, these should be maintained.

The indicator *maternal disorders* obtained less than 85% agreement among the judges and a p-value <0.05 in the binomial test and, therefore, should be removed. One judge suggested its removal because this indicator is not considered strong in relation to developmental delay. The indicator for eating disorders obtained a concordance index of less than 85% and a p-value >0.05, indicating that there is no statistical significance and that it should be retained. One judge suggested that this indicator should be reviewed together with the indicator for malnutrition, as the latter falls within the category of eating disorders. The indicator *infections* obtained a concordance index of less than 85% and a value of p > 0.05, indicating that there is no statistical significance and that it should be maintained. It was suggested that it be reformulated to untreated infections. It was also suggested that the following factors be added to this indicator: ZIKA virus, HIV, syphilis, and rubella.

During the consensus analysis, most of the elements were maintained and only “malformation” was modified to be included within congenital disorders; “Vitamin B12 deficiency” should be inserted in “Eating disorders” and this should change the nomenclature to “Nutritional disorders.”

### Evaluation of Related Factors and Defining Characteristics

Table 3 shows the Related Factors (RF) and Defining Characteristics (DC) according to the judges’ agreement index.

**Table 3:** Judges' index of agreement regarding related factors and defining characteristics – Redenção, CE, Brazil, 2022.

Related factors	n	%	p
Difficulty eating	20	91	0.338
Mistreatment	19	86	0.575
Altered sleep pattern	21	95	0.137
Mothers**’** inadequate knowledge of ID	21	95	0.137
**Defining characteristics**	**n**	**%**	**p**
Cognitive delay	22	100	0.028
Learning delay	22	100	0.028
Delayed problem solving	21	95	0.137
Language delay	22	100	0.028
Receptive language delay	22	100	0.028
Expressive language delay	22	100	0.028
Lack of verbal communication skills	21	95	0.137
Psychosocial delay	22	100	0.028
Socio-emotional problems	22	100	0.028
Socialization problems	16	73	0.100
Behavioral deficiency	20	91	0.338
Challenging behavior	20	91	0.338

As for Related Factors, it is possible to see that all items had an agreement rate of over 85% among the judges. Only the element “Abuse” was suggested to be removed from the RF and inserted into Populations at Risk, which was therefore accepted. During the consensus analysis, all experts reaffirmed the importance of retaining the same RFs and adding a new one, entitled “Use of formula in children under six months of age.”

In the defining characteristics, although all elements were well evaluated in the agreement validation, it was still suggested that some be unified because they were similar, such as “Lack of verbal communication skills,” which was removed because it was already covered in “Language delay.” The elements “Socioemotional problems” and “Behavioral impairment” were also removed because they belonged to other elements.

In the consensus validation, it was recommended that all defining characteristics be renamed in order to adapt them to the structure and terminology used in nursing diagnoses, facilitating understanding and applicability in clinical practice. Thus, the terms “Cognitive delay” and “Learning delay” were unified under the name “Impaired cognitive ability in learning acquisition.” Similarly, “Cognitive delay” and “Delay in problem solving” were consolidated into “Cognitive difficulty in problem solving.” The term “language delay” was given an age range specification and is now described as “language delay in infants.” The expressions “Receptive language delay” and “Expressive language delay” were reformulated to “Infant expresses difficulty in receptive language” and “Infant expresses difficulty in expressive language,” respectively. In addition, the terms “psychosocial delay” and “socialization problems” were renamed “difficulty in social interaction” and, finally, “challenging behavior” was renamed “displays behavior that is not socially acceptable or desirable.” However, the latter was suggested for removal, as it was not related to children in the age group up to 2 years old. At first, it also received a low score. Therefore, it was accepted.

The Chart 1 below shows all the structural elements of the nursing diagnosis Delayed Infant Development, after the content analysis phase by the nurse judges.

**Chart 1:** Elementos estruturais do diagnóstico de enfermagem Desenvolvimento Atrasado do Lactente, após a fase de análise de conteúdo pelos enfermeiros juízes – Redenção, CE, 2025.

Title
Delayed Infant Development
**Definition**
Infants show an absence of two or more milestones in areas of development, characterized by cognitive, communication/language, and personal/social changes
**Related factors**
– Feeding difficulties – Changes in sleep patterns – Inadequate knowledge of parents/caregivers about infant development - Use of formula in children under 6 months of age
**Defining characteristics**
– Impaired cognitive ability in learning acquisition – Impaired cognitive ability in problem solving – Delayed language development in infants – Infants express difficulty in receptive language – Infants express difficulty in expressive language – Difficulty in social interaction
**Population at risk**
– Genetic factors: male gender; black population; – Disadvantaged social factors: disadvantaged paternal occupation; economically disadvantaged family; lack of access to early childhood education; limited variety of toys; social isolation of the mother during the child's early years; environmental conditions (housing, water supply, presence of septic tanks and sewers); – Unfavorable family environment: parental stress; low stimulation; low maternal and caregiver education; presence of two or more siblings; missing family member, especially the mother; first-degree relatives with language delays; low father-child interaction; – Unfavorable perinatal outcomes: prematurity; low birth weight; short stature; low and high head circumference; – Unfavorable obstetric outcomes: age < 20 years; single mothers; multiparity; substance abuse during pregnancy (alcohol, tobacco, and other drugs); unwanted pregnancy; infants exposed to HIV and other sexually transmitted infections; prenatal exposure to air pollutants - NO2 and benzene; – Child abuse (physical, exposure to violence, and neglect).
**Associated conditions**
Malnutrition; invasive procedures (cesarean section and forceps delivery, children undergoing heart surgery, admission to neonatal intensive care); prolonged exposure to medications (dexamethasone, hydrocortisone in premature infants, anesthetics, atypical antipsychotics in the prenatal period, oxygen therapy); prenatal and postnatal factors (venous thrombosis of the placenta, intrauterine growth restriction, maternal obesity, gestational diabetes and maternal malnutrition, pre- and postpartum depression, postpartum anxiety, maternal mental disorder, untreated prenatal infections); neonatal disorders (5-minute Apgar score <3, bronchopulmonary dysplasia, perinatal asphyxia with hypoxic ischemic encephalopathy, peri – intraventricular hemorrhage, sequelae of prematurity); neurological disorders (childhood epilepsy, cerebral palsy, mental retardation); genetic disorders (Down syndrome, Myrhe syndrome, Williams syndrome); congenital disorders (congenital heart disease, congenital profound visual impairment, malformation); untreated neonatal infections (otitis; neonatal sepsis; neonatal infections: cytomegalovirus, Zika virus, HIV, syphilis, and rubella); linear and weight growth retardation; attention deficit hyperactivity disorder; autism; hearing loss; head trauma; metabolic disorders; nutritional disorders: vitamin B12 deficiency.

## DISCUSSION

The proposed nursing diagnosis showed high agreement among the judges regarding the relevance of its components, which were classified as relevant, obtaining a percentage of agreement greater than 85% in almost all items, which supports the content validity of this diagnosis. Thus, this result shows that these elements are aligned with clinical practice and theoretical knowledge about child development, especially in the infant stage.

The judges or experts were mostly female and held doctoral degrees. It was noted that the highest response rate was from nurses working in the area of child health. However, when searching for these professionals, an effort was made to balance those with experience in child health and those with experience in nursing taxonomies. This makes the evaluation of the elements of the proposed diagnosis more accurate, contextualized, and coherent, in addition to ensuring that they are aligned with nursing practice and standardized nursing language^(11)^.

Moving on to content analysis, the title and definition of the proposed diagnosis were well accepted by the judges in the agreement analysis. However, during the consensus analysis, the experts suggested modifying the way they were written so that they resembled the nomenclature of nursing taxonomy, especially that used by NANDA-I, which will be used for the diagnosis. This was done in order to standardize the writing, as this could contribute to effective communication between professionals and continuity of care^(12)^.

At-risk populations refer to groups of people who share characteristics that make each of them susceptible to a particular human response. In the proposed diagnosis, five groups of at-risk populations were listed with their respective components. One of them with the greatest disagreement among the judges was “Genetic factors,” which covers males and the black population. However, it was maintained because there are studies that show that this gender is more likely to experience developmental delays^(13)^. In addition, the black population should also be included as a factor that is directly related to developmental delays, due to the significant challenges faced by this population, such as racism, social inequalities, and limited access to essential services^(14,15)^.

In the “Social Factors” section, also belonging to Populations at Risk, environmental conditions, housing, water, supply, and the presence of septic tanks and sewers were added, as it is known that expanding access to basic sanitation is a determining factor in reducing damage to children’s health^(16)^. Thus, we can consider that infants living in unfavorable environmental and housing conditions, with a lack of water treatment and supply and a lack of septic tanks, live in conditions that put them at risk for developmental delays^(17)^. Thus, their inclusion, validated by experts as relevant, reinforces the applicability of the diagnosis in different contexts, especially in regions with less access to more complex diagnostic resources.

In the associated conditions, a broad list was compiled, grouped with components belonging to them, **and** identified as multiple situations in which nurses need other professionals to intervene in order to obtain favorable results, that is, conditions that are not independently modifiable by nurses, but which it is necessary to be aware of. In the present study, conditions such as genetic, neurological, and metabolic disorders were listed, which are important in compromising child neurodevelopment and, although they cannot be directly modified by nursing, constitute clinical alerts for early screening and systematization of care by these professionals^(4)^. In addition, the literature states that validated nursing diagnoses are tools that facilitate the adequate monitoring of these conditions, allowing nurses to act in a coordinated manner and in partnership with other professionals^(18)^.

The related factors were considered relevant and well accepted in both evaluations. Only the component “Abuse” was redirected to the associated conditions, as it refers to exposure to a specific event and is therefore not part of the RF. In addition to the addition of a new element, “Use of formula in children under six months of age,” its inclusion is in line with a bibliographic review study, which demonstrated in its results articles that showed that babies who were exclusively breastfed until six months of age had better neuropsychomotor development than those who were breastfed until four months of age, or even those who were not breastfed in the first year of life^(19)^. Other factors also deserve mention, such as “Inadequate knowledge of caregivers” and “changes in sleep patterns,” as they are recognized in the literature as environmental modifiers of development. Based on these factors, the role of nurses in conducting health education activities and supporting responsive parenting practices is reinforced, with the aim of intervening or even preventing developmental delays^(20)^.

The Defining Characteristics (DC) of the diagnosis “Delayed Infant Development” include a set of signs that will be present when the child shows developmental delays. In this proposal, seven DCs were ultimately defined for different domains, such as cognition, language, and personal-social. During the consensus analysis, it was necessary to reformulate the terminology, following the NANDA-I standardization, in order to facilitate communication between professionals and operationalize the diagnosis in different care contexts. In addition, the existence of multiple DCs increases diagnostic accuracy, according to Fehring^(7)^, thus strengthening more targeted care plans.

Thus, the DCs related to cognition and language (impaired cognitive ability in learning and problem solving, infant language delay, and infant, expressing difficulties in receptive and expressive language) are aligned with the international literature, which recommends systematic observation of developmental milestones related to these domains in order to detect delays early^(21)^. In addition, it is necessary to pay attention to the monitoring of psychosocial aspects, such as in the case of the DC “Difficulty in social interaction,” because once identified, this child may face challenges related to understanding, social responses, and effective communication, aspects found in children with neurodevelopmental disorders^(22)^.

Therefore, identifying these DCs will allow nurses to develop interventions aimed at promoting the development of these skills in partnership with a multidisciplinary team.

The limitations of this study refer to the analysis performed only with professionals in the field of child health and/or nursing taxonomies, which may limit the diversity of perspectives on the proposed diagnosis. On the other hand, it increases the specificity of the topic. Another limitation is the lack of practical application in real clinical contexts, requiring future validation with the target audience to strengthen the applicability of the diagnosis in nursing care.

## CONCLUSION

The proposed nursing diagnosis “Delayed Infant Development” presented satisfactory content validity, with high agreement among the judges regarding its components, including title, definition, related factors, defining characteristics, at-risk populations, and associated conditions. The reformulation of the nomenclature of defining characteristics in accordance with NANDA-I language linked to scientific evidence allows this diagnosis to be configured as an applicable, useful, and sensitive tool for clinical nursing practice. In addition to enabling the early recognition of developmental delays in a sensitive age group, such as infants.

## Data Availability

The entire dataset supporting the results of this study is available upon request to the corresponding author.
