# Supplementary material for: Delayed infant development: content analysis of a nursing diagnosis proposal
Source: Rev Esc Enferm USP. 2026 May 15;60:e20250400. doi: 10.1590/1980-220X-REEUSP-2025-0400en (PMC13178669; doi:10.1590/1980-220X-REEUSP-2025-0400en)
Supplement: Translation

# Translation: Desenvolvimento atrasado do lactente: análise de conteúdo de uma proposta de diagnóstico de Enfermagem*

**DOI:** 10.1590/1980-220X-REEUSP-2025-0400pt

**Authors:** Maria do Socorro Távora de Aquino, Samara Pereira Souza Mariano, Flávia Paula Magalhães Monteiro, Huana Carolina Cândido Morais, Vanessa Emille Carvalho de Sousa Freire, Maria Nataniele Queiroz de Lima, Quéren-Hapuque Lopes Sousa

**Affiliations:** 1 Universidade da Integração Internacional da Lusofonia Afro-Brasileira, Instituto Ciências da Saúde, Redenção, CE, Brasil.

**Keywords:** Diagnóstico de Enfermagem, Deficiências do Desenvolvimento, Estudos de Validação

## Abstract

**Objetivo:**

Analisar o conteúdo do diagnóstico de Enfermagem “Desenvolvimento atrasado do lactente”.

**Método:**

Estudo metodológico, abordando-se a fase de análise de conteúdo, valendo-se de uma abordagem mista. A primeira etapa foi um estudo de concordância, quantitativo; a segunda, um estudo de consenso qualitativo. As duas etapas foram realizadas com proficientes. Para análise, empregou-se o teste Binomial, o valor p igual ou superior a 0,05 com ponto de corte de 0,85. O nível de significância adotado foi de 5%.

**Resultados:**

O diagnóstico proposto obteve elevada concordância entre os juízes quanto à sua estrutura (título, definição, características definidoras, fatores relacionados, populações em risco e condições associadas), com mais de 85% de aprovação. Algumas características foram reformuladas para alinhamento com a NANDA-I, aprimorando a clareza e a aplicabilidade clínica.

**Conclusión:**

A validade de conteúdo foi satisfatória, mostrando-se relevante, clara e aplicável à prática de Enfermagem. Sua adoção pode favorecer a detecção precoce de atrasos no desenvolvimento infantil e subsidiar intervenções multiprofissionais, especialmente em contextos de vulnerabilidade.

## INTRODUÇÃO

O desenvolvimento infantil (DI) abrange o espectro neuropsicomotor e pode ser dividido, resumidamente em domínios, dentre os quais se destacam o cognitivo, o psicossocial e o físico, que estão interligados e se influenciam mutuamente durante a vida do indivíduo^(1)^. Dessa forma, alterações nesses domínios podem levar ao atraso no desenvolvimento. Porém, para que sejam detectadas tais alterações, a criança deve passar por vigilância contínua, a fim de detectar problemas precocemente, ou mesmo acompanhar o atraso quando este já estiver instalado, por meio de instrumentos padronizados e validados^(2)^.

Todavia, a tarefa de identificar e acompanhar crianças com atraso no desenvolvimento neuropsicomotor depara-se com a complexidade de fatores, como a inexistência de um sistema eficiente de vigilância, a não utilização de instrumentos padronizados e adequados para realização da triagem ou mesmo a não gratuidade de testes que são considerados padrão-ouro na avaliação do atraso do desenvolvimento^(3)^.

O período em que esses fatores influenciarão mais fortemente dependerá, também, de cada fase/etapa designadas de acordo com o ciclo vital da criança e sua idade. Conforme as faixas etárias consideradas pelo Ministério da Saúde (MS), é na fase de lactente, que compreende a idade de 29 dias até 24 meses, que ocorrem as maiores e mais rápidas modificações no DI, em decorrência da maior plasticidade cerebral. Consequentemente, as crianças que estão nessa fase também estão expostas a agravos, como atrasos no DI. No entanto, elas também possuem uma melhor respostas a terapias e estímulos, quando diagnosticadas precocemente^(4)^.

O enfermeiro é um dos profissionais que pode fazer a triagem do DI, através da utilização dos diagnósticos de Enfermagem no intuito de direcionar suas condutas. Contudo, não há um diagnóstico de Enfermagem que trabalhe especificando cada faixa etária e, como elencado acima, há uma faixa etária, em específico, mais sensível a diagnóstico e intervenção para o DI, a fase de lactente. A partir disso, um estudo anterior^(5)^ construiu os elementos estruturais de um diagnóstico de Enfermagem voltado para o atraso no desenvolvimento do lactente. Todavia, ainda não havia realizado a etapa de análise de conteúdo, que é primordial para o processo de validação final do diagnóstico.

Essa análise de conteúdo refere-se a uma das etapas para construção ou validação de diagnóstico de Enfermagem, que tem como objetivo examinar criticamente os seus elementos estruturais, como título, definição, fatores relacionados, características definidoras, populações em riscos e condições associadas, através de critérios psicométricos e participação de juízes especialistas, que garantam que esses componentes estejam coerentes com a prática clínica e com uma linguagem padronizada da Enfermagem, como a da NANDA-Internacional^(6)^.

Vale destacar, ainda, que a utilização de diagnósticos de Enfermagem validados permite a elaboração de planos assistenciais mais eficazes, auxiliando na promoção da saúde infantil e prevenção de agravos. Contudo, para que essa ferramenta seja aplicada de maneira eficiente, é necessário que seu conteúdo seja previamente analisado e avaliado por especialistas, garantindo sua pertinência e clareza na prática clínica.

Diante disso, o objetivo desse estudo foi analisar o conteúdo do diagnóstico de Enfermagem “Desenvolvimento atrasado do lactente”, utilizando uma abordagem mista.

## MÉTODO

### Desenho do Estudo

Estudo metodológico, abordando-se a fase de análise de conteúdo, valendo-se de uma abordagem mista, com delineamento exploratório sequencial. A primeira etapa foi um estudo de concordância, quantitativo, e a segunda, um estudo de consenso, qualitativo. As duas etapas foram realizadas com proficientes na área. Contudo, na segunda houve um rigor maior ao selecionar esses juízes, sendo considerados, principalmente, aqueles com doutorado e que trabalhassem/pesquisassem na área materno-infantil e/ou taxonomias de Enfermagem. Isso ocorreu pois eles iriam tanto avaliar as discordâncias da primeira etapa, quanto reavaliar itens que julgassem pertinentes.

### Local e Período

O estudo ocorreu de forma remota. A primeira etapa ocorreu entre novembro de 2018 a janeiro de 2019, de maneira assíncrona, através de contato via e-mail. A segunda etapa foi realizada entre outubro de 2020 a janeiro de 2021, e ocorreu tanto de forma assíncrona, também por e-mail, como síncrona, pela plataforma *Google Meet*.

### População, Amostra e Critérios de Seleção

Nas duas etapas, a captação dos juízes/expertises ocorreu por meio de busca na Plataforma Lattes do Currículo Lattes de pesquisadores, disponível no portal do Conselho Nacional de Desenvolvimento Científico e Tecnológico (CNPq) utilizandose os seguintes termos: análise de conceito; diagnósticos de enfermagem; crescimento e desenvolvimento infantil; saúde da criança; análise de conteúdo e validação de conteúdo e por meio de amostragem de rede ou bola de neve (*snowball*), durante a qual foi solicitada indicação de outro pesquisador que pudesse atender aos critérios de elegibilidade deste estudo e o convite era feito através do e-mail de cada juiz.

A fim de considerar tal juiz apto para realizar a avaliação, foram aplicados os critérios de Fehring^(7)^, de forma adaptada, e consideradas também as recomendações de Lopes et al.^(8)^, que mencionam o equilíbrio dos participantes quanto à experiência clínica e o conhecimento teórico. Logo depois da seleção, estes foram convidados e divididos por perfil: o primeiro, formado por enfermeiros clínicos com experiência de pelo menos cinco anos trabalhando na área de saúde da criança e desenvolvimento infantil; e o segundo, formado por juízes com experiência com o diagnóstico de enfermagem e taxonomia da NANDA Internacional (NANDA-I).

Dessa forma, para análise de concordância (1ª etapa), o tamanho amostral foi definido por meio do cálculo que considera a proporção esperada de sujeitos em relação a uma variável dicotômica e a diferença máxima aceitável dessa proporção. O cálculo possui a fórmula: n = Zα2.P.(1-P)/d2, em que Zα refere-se ao nível de confiança no intervalo, adotado em 95%, correspondendo ao valor tabelado de 1,96; P é a proporção de indivíduos que concordam com a pertinência do componente do diagnóstico de enfermagem, sendo considerada de 85%; e d é a diferença de proporção considerada aceitável, que será em 15%, incluindo um intervalo de 70 a 100%. Desse modo, o cálculo final foi determinado por n = 1,962.0,85.0,15/0,152, sendo concluído um número amostral de 22 juízes^(8)^.

Para este momento, foram recrutados 135 enfermeiros, no primeiro contato. Obteve-se o retorno de 36 juízes, sendo que dentre estes, cinco recusaram a participar do estudo por motivos acadêmicos e profissionais e nove, embora inicialmente tenham aceitado, não devolveram a avaliação dentro do prazo estabelecido, sendo, portanto, excluídos da amostra. Assim, a amostra final foi composta por 22 enfermeiros juízes.

Já a segunda etapa do estudo foi denominada como análise de consenso, pois se buscou seguir princípios metodológicos característicos desse tipo de abordagem^(9)^. A exemplo da definição dos critérios para seleção dos avaliadores, já mencionados acima, adotou-se ainda, a estratégia de discussão em grupos com o objetivo de alcançar consenso entre os participantes acerca dos itens do diagnóstico. O processo envolveu rodadas sucessivas de feedback e discussão coletiva, nas quais os comentários e sugestões dos juízes foram considerados para a revisão e aperfeiçoamento dos itens propostos, caracterizando, assim, uma etapa de validação por consenso entre especialistas.

A amostragem foi não-probabilística, através do grupo focal (GF). Neste tipo de abordagem, determina-se o número de juízes entre seis e 15. Foram recrutados 61 enfermeiros, no primeiro contato, e obteve-se o retorno de 15 juízes, sendo que oito recusaram-se a participar do estudo por motivos acadêmicos e profissionais. A amostra final foi constituída por sete enfermeiros juízes.

### Coleta de Dados

Após aceitarem participar no estudo, cada juiz recebeu, via e-mail, o Termo de Consentimento Livre e Esclarecido (TCLE) e o questionário para avaliação elaborado na fase de análise de conceito, com os elementos do diagnóstico proposto “Desenvolvimento atrasado do lactente”, contendo título, definição, características definidoras, fatores relacionados, populações em risco e condições associadas. Para o estabelecimento do consenso entre os juízes, com o intuito de nortear os indicadores a serem analisados no instrumento, foi utilizada a relevância como um dos critérios de psicometria propostos por Pasquali (1999)^(10)^, sendo estabelecida uma escala do tipo Likert com 5 opções de respostas, onde os itens 1, 2 ou 3 são classificados como inadequados/irrelevantes; já os itens 4 e 5 são considerados adequados/relevantes.

No intuito de estabelecer o consenso entre os juízes de forma coletiva, foram realizadas reuniões via plataforma *Google Meet*, estabelecendo-se a metodologia do GF. Foram realizados dois encontros, com duração de duas horas cada. O grupo foi conduzido por um mediador, um dos pesquisadores do estudo, e os dois momentos foram gravados para posterior registro e análise. Esses mesmos juízes já haviam avaliado o instrumento, previamente, de forma individual, e os dados da avaliação prévia foram apresentados ao GF pelo mediador e discutido por ambos. Todos os questionamentos e sugestões foram discutidos até a obtenção do consenso, sendo este considerado quando a maioria do grupo concordava com a síntese da discussão sobre cada questionamento e sugestão realizada pelo mediador.

### Análise e Tratamento dos Dados

Os dados foram compilados no programa Excel® 2016 e, após, para a tabulação, cálculo das médias e testes estatísticos, foi utilizado o programa *Statistical Package for the Social Sciences* (SPSS), versão 25.0.

A análise estatística de concordância, segundo cada item do instrumento, foi realizada por meio da adequação do ajustamento das proporções dos juízes que concordaram com a pertinência do instrumento. Para tanto, empregou-se o teste Binominal, sendo pertinente o valor p igual ou superior a 0,05 com ponto de corte de 0,85. Para essa análise, o nível de significância (α) adotado foi de 5%.

### Aspectos Éticos

Foram cumpridos os preceitos da Resolução 466/2012 do Conselho Nacional de Saúde que trata da pesquisa com seres humanos. O projeto foi registrado na Plataforma Brasil e aprovado pelo CEP/UNILAB, protocolo no. 5.032.639.

## RESULTADOS

Para o estudo de concordância, os enfermeiros apresentaram taxa de retorno dos instrumentos de 16,2%. Destes, 95,5% (n = 21) eram do sexo feminino, 59,1% (n = 13) trabalhavam em Instituição de Ensino Superior, 63,3% (n = 14) com titulação de doutor e 36,4% (n = 08) com especialização na área de saúde da criança. Sobre a atuação na linha de pesquisa em saúde da criança, 77,3% (n = 17) mencionaram que já haviam atuado e/ou atuavam e 45,5% (n = 10) apresentavam expertise na área de diagnóstico de enfermagem.

As variáveis idade e tempo de formação apresentaram distribuição assimétrica (p < 0,05), indicando que a mediana da idade foi 33,50 (±8,78) anos, sendo a idade mínima 27 anos, e a máxima de 59 anos. Quanto ao tempo de formação, a mediana foi de 10 (±7,21) anos, sendo o tempo mínimo de cinco anos e máximo de 35 anos.

Já no estudo de consenso, todas as participantes eram do sexo feminino. Quanto à titulação, duas (28,6%) enfermeiras possuíam especialização na área de desenvolvimento infantil, uma em neonatologia e todas (100%) eram doutoras. Além disso, o tempo de formação variou entre 10 e 20 anos. Todas estavam trabalhando como docentes em universidades de diversos estados, como Ceará, Paraíba, Bahia, Piauí, Goiás e São Paulo e trabalhavam temas relacionados à saúde da criança e/ou taxonomias de Enfermagem.

Os resultados acerca da avaliação de conteúdo e consenso dos elementos do diagnóstico proposto serão organizados em tabelas pelos seguintes tópicos: avaliação de título e definição, avaliação da população em risco e condições associadas, avaliação das características definidoras e avaliação dos fatores associados.

### Avaliação de Título e Definição

Para análise de conteúdo, foram enviadas uma opção de título do diagnóstico e três definições construídas previamente durante a análise de conceito, e as opções de justificativa e de sugestão, caso considerassem necessárias. Ressalta-se que, na etapa de análise de conceito foi realizada leitura minuciosa dos artigos selecionados para construção das definições.

Na Tabela 1, encontra-se o julgamento quanto à adequação do título do diagnóstico de enfermagem proposto e sua respectiva definição final escolhida pelos juízes.

**Tabela 1:** Avaliação da adequação do título e definição para o diagnóstico de enfermagem proposto – Redenção, CE, Brasil, 2022.

Elementos	n	%
**Título:** Atraso do desenvolvimento do lactente.	22	100
**Definição:** “Atraso significativo de duas ou mais áreas do desenvolvimento, caracterizadas por alterações que podem incluir o desempenho motor (grosso ou fino), comunicação/linguagem, cognitivo, social e atividades da vida diária. O atraso se configura por alterações ≥ 2 desvios-padrão abaixo da média”.	20	90,9

É possível observar que houve grande concordância nos dois elementos apresentados. Ainda assim, as expertises da análise de consenso sugeriram algumas modificações quanto a escrita para ser melhor aceita pela taxonomia NANDA-I durante a sua submissão. Dessa forma, o título passou a ser “Desenvolvimento Atrasado do Lactente” e a definição: “Lactente apresenta ausência de dois ou mais marcos nas áreas do desenvolvimento, caracterizadas por alterações cognitivas, comunicação/linguagem e pessoal/social”, justificando-se por ser mais completa ao trazer os domínios que poderão ocorrer o atraso. Vale destacar que todas essas alterações foram acatadas por se tratar de profissionais com ampla experiência nessa área e que estavam avaliando em conjunto, sendo consenso entre todos essas modificações.

### Avaliação da Adequação das Populações em Risco e Condições Associadas

A seguir, na Tabela 2 são apresentados os indicadores que compuseram as populações em risco e condições associadas por meio da análise de conceito, segundo o julgamento mediante o índice de concordância dos juízes quanto ao critério da relevância.

**Tabela 2:** Índice de concordância dos juízes quanto à relevância dos indicadores das populações em risco e condições associadas – Redenção, CE, Brasil, 2022.

Populações em risco	n	%	p^*^
Fatores sociais (Ocupação paterna desfavorecida; Família economicamente desfavorecida; Falta de acesso à educação infantil; Crianças que vivem em países em desenvolvimento; pouca variedade de brinquedos; Isolamento social da mãe nos primeiros anos de vida da criança)	22	100	0,028
Ambiente familiar desfavorável (Estresse parental; Baixo estímulo; Baixa escolaridade materna; Cuidadores sem ensino superior; Presença de 2 ou mais irmãos; Família faltando membro, principalmente a mãe; Parentes de 1° grau com atraso na linguagem; Baixa interação pai-filho)	22	100	0,028
Condições neonatais (Prematuridade; Baixo peso; Baixa estatura; Baixo Perímetro cefálico)	22	100	0,028
Condições obstétricas (Idade < 20 anos; Mães solteiras; Multiparidade; Abuso de substâncias na gestação (álcool, tabaco e outras drogas); Gravidez não-desejada; lactentes expostos ao HIV; Exposição pré-natal a poluentes do ar - NO2 e benzeno)	21	95	0,137
Fatores genéticos (Sexo masculino; Etnia não-branca)	13	59	0,003
**Condições associadas**	**n**	**%**	p^*^
Exposição a medicamentos (Uso neonatal de Dexametasona; Tratamento prolongado com Hidrocortisona em prematuros; Exposição a anestésicos por tempo prolongado; Exposição aos antipsicóticos atípicos no período pré-natal)	22	100	0,028
Distúrbios neonatais (Apgar de 5 minutos <3; Displasia broncopulmonar; Asfixia perinatal com encefalopatia isquêmica hipóxica; Hemorragia peri-intraventricular; Sequelas da prematuridade)	22	100	0,028
Distúrbios neurológicos (Epilepsia infantil; Paralisia cerebral; Retardo mental)	22	100	0,028
Distúrbio genético (Sd. de Down; Sd. de Myrhe; Sd. de Williams)	22	100	0,028
Distúrbios congênitos (Doença cardíaca congênita; Deficiência visual profunda congênita)	22	100	0,028
Trauma cefálico	22	100	0,028
Distúrbios metabólicos	22	100	0,028
Má formação (Cerebral; Craniossinostose; Lábio leporino)	22	100	0,028
Transtorno do Déficit de Atenção com Hiperatividade (TDAH)	21	95	0,137
Autismo	21	95	0,137
Perda auditiva	21	95	0,137
Fatores pré-natais (Trombose venosa da placenta; RCIU; Obesidade materna; Diabetes gestacional)	21	95	0,137
Desnutrição infantil	20	91	0,338
Infecções (Lactentes infectados por Citomegalovírus; Otite; Sepse neonatal; Infecções neonatais)	18	82	0,425
Procedimentos (Parto cesárea; Criança submetida à cirurgia cardíaca; Admissão em cuidados intensivos neonatais; Oxigenioterapia)	17	77	0,226
Atraso no crescimento	17	77	0,028
Deficiência de vitamina B12	17	77	0,226
Distúrbios alimentares	17	77	0,226
Distúrbios maternos (Depressão pré-natal e pós-parto; Ansiedade pós-parto; Distúrbio psíquico materno)	16	73	0,000

*Teste binomial.

Considerando que a hipótese nula seja representada pelos valores de concordância entre os juízes iguais a 85%, o indicador das populações em risco relacionado às condições genéticas é significativo com relação a não concordância entre os juízes, e assim, deve ser retirado. Ainda sobre esse indicador, um juiz sugeriu separar os seus componentes (sexo masculino e etnia não-branca) em grupos diferentes, não os associando com condições genéticas.

Os demais indicadores tiveram índice de concordância maior que 85% entres os juízes e o valor de p> 0,05 pelo teste binomial, e assim, foram aprovados pelos juízes. Contudo, houve algumas sugestões, como: nos “Fatores sociais” foi sugerido acrescentar a eles os elementos, condições ambientais, moradia, água, abastecimento e presença de fossas sépticas e esgotos. Em “condições neonatais”, sugerido acrescentar alto perímetro cefálico. Nas condições obstétricas, inseriu o termo Infecções Sexualmente Transmissíveis (IST), além da exposição pelo vírus HIV no lactente. Já na análise de consenso, as expertises mantiveram as respostas. Frisaram, ainda, a importância de cada elemento ter seus componentes associados. Além da modificação do termo “etnia não-branca” para “população negra” dentro dos fatores genéticos. Não concordando, portanto, em seu desmembramento, como sugerido anteriormente.

No que concerne às Condições Associadas, o indicador *desnutrição infantil* obteve concordância entre os juízes maior que 85%, sendo sugerido a retirada do termo “infantil”. Além disso, os indicadores, *procedimentos, atraso no crescimento, malformação, deficiência de vitamina B12, distúrbios alimentares e infecções* obtiveram índice de concordância menor que 85%. Contudo, para o teste binomial, estes devem ser mantidos.

Já o indicador *distúrbios maternos* obteve concordância menor que 85% entre os juízes e valor de p < 0,05 no teste binomial e, portanto, deve ser retirado. Um juiz sugeriu sua retirada pelo fato desse indicador não ser considerado forte quanto à relação com o atraso do desenvolvimento. Em relação ao indicador *distúrbios alimentares* obteve índice de concordância menor que 85% e valor de p > 0,05, indicando que não há significância estatística e que deve ser mantido. Um juiz sugeriu que este indicador deve ser revisado juntamente com o indicador desnutrição, pois este último se insere dentro dos distúrbios alimentares. O indicador *infecções* obteve um índice de concordância menor que 85% e valor de p > 0,05, indicando que não há significância estatística e que deve ser mantido. Foi sugerido sua reformulação para infecções não-tratadas. Ainda foi sugerido acrescentar como fatores deste indicador: ZIKA vírus, HIV, sífilis e rubéola.

Já durante a análise de consenso, foi mantida a maioria dos elementos e modificado apenas a “má formação”, para ser incluída dentro dos distúrbios congênitos; a “Deficiência de vitamina B12”, deveria ser inserida nos “Distúrbios alimentares” e este modificasse a nomenclatura para “Distúrbios nutricionais”.

### Avaliação dos Fatores Relacionados e Características Definidoras

Na Tabela 3, são apresentados os Fatores Relacionados (FR) e Características Definidoras (CD), segundo o julgamento mediante o índice de concordância dos juízes.

**Tabela 3:** Índice de concordância dos juízes quanto aos fatores relacionados e as características definidoras – Redençã, CE, Brasil, 2022.

Fatores relacionados	n	%	p
Dificuldade na alimentação	20	91	0,338
Maus-tratos	19	86	0,575
Alteração no padrão de sono	21	95	0,137
Conhecimento inadequado das mães sobre DI	21	95	0,137
**Características definidoras**	**n**	**%**	**p**
Atraso cognitivo	22	100	0,028
Atraso na aprendizagem	22	100	0,028
Atraso na resolução de problemas	21	95	0,137
Atraso na linguagem	22	100	0,028
Retardo na linguagem receptiva	22	100	0,028
Retardo na linguagem expressiva	22	100	0,028
Ausência de habilidades na comunicação verbal	21	95	0,137
Atraso psicossocial	22	100	0,028
Problemas socioemocionais	22	100	0,028
Problemas de socialização	16	73	0,100
Deficiência comportamental	20	91	0,338
Comportamento desafiador	20	91	0,338

Quanto aos Fatores Relacionados, é possível perceber que todos os itens apresentaram uma concordância maior que 85% entre os juízes. Apenas ao elemento “Maus-tratos” foi sugerido para ser retirado dos FR e inserido nas Populações em Risco, sendo, portanto, acatado. Já durante a análise de consenso, todas as expertises reafirmaram a importância de permanecer com os mesmos FR e inserir um novo, sendo intitulado “Utilização de fórmula na criança menor de seis meses”.

Nas características definidoras, apesar de todos os elementos terem sido bem avaliados na validação de concordância, ainda assim, foi sugerida a unificação de alguns por se assemelharem, a exemplo de “Ausência de habilidades na comunicação verbal”, que foi retirado por já estar contemplado em “Atraso na linguagem”; também os elementos “Problemas socioemocionais” e “deficiência comportamental” foram retirados, por pertencerem a outros elementos.

Já na validação de consenso, recomendou-se a renomeação de todas as características definidoras, com o objetivo de adequálas a estrutura e terminologia utilizadas nos diagnósticos de Enfermagem, facilitando a compreensão e aplicabilidade na prática clínica. Assim, os termos “Atraso cognitivo” e “Atraso na aprendizagem” foram unificados sob a denominação “Capacidade cognitiva prejudicada na aquisição da aprendizagem”. Da mesma forma, “Atraso cognitivo” e “Atraso na resolução de problemas” foram consolidados em “Dificuldade cognitiva na resolução de problemas”. O termo “Atraso na linguagem” recebeu a especificação de faixa etária, passando a ser descrito como “Atraso na linguagem do lactente”. As expressões “Atraso na linguagem receptiva” e “Atraso na linguagem expressiva” foram reformuladas para “Lactente expressa dificuldade na linguagem receptiva” e “Lactente expressa dificuldade na linguagem expressiva”, respectivamente. Além disso, os termos “Atraso psicossocial” e “Problemas de socialização” passaram a ser denominados como “Dificuldade na interação social” e, por fim, “Comportamento desafiador” passou a ser chamado “Apresenta comportamento não aceitável ou desejado socialmente”. Contudo, este último foi sugerido sua retirada, por não apresentar relação com as crianças na faixa etária de até 2 anos. No primeiro momento também atingiu uma pontuação baixa. Sendo, portanto, acatado.

Os juízes ou expertises eram, em sua maioria, do sexo feminino e com título de doutor. Percebeu-se que a taxa de retorno maior foi dos enfermeiros que atuavam na área de saúde da criança. Porém, na realização da procura por esses profissionais, buscou-se equilibrar aqueles que possuíam experiência na área de saúde da criança e na área das taxonomias de Enfermagem. Dessa forma, a avaliação dos elementos do diagnóstico proposto se torna mais precisa, contextualizada e coerente, além de assegurar que eles estejam alinhados à prática assistencial e à linguagem padronizada da Enfermagem^(11)^.

Partindo para a análise de conteúdo, o título e a definição do diagnóstico proposto foram bem aceitos pelos juízes na análise de concordância. No entanto, para as expertises, durante a análise de consenso, sugeriram modificar a forma de escrevêlos, para que se assemelharam à nomenclatura da taxonomia de Enfermagem, especialmente a utilizada pela NANDA-I, a qual o diagnóstico será proposto. Isto ocorreu no intuito de padronizar essa escrita, pois a partir disso, poderá contribuir para a comunicação eficaz dos profissionais e continuidade do cuidado^(12)^.

As populações em risco se referem a grupos de pessoas que compartilham características que levam cada uma delas a ser suscetível a determinada resposta humana. No diagnóstico proposto foram elencados cinco grupos das populações em risco com seus respectivos componentes. Um deles com maior discordância entre os juízes foram os “Fatores genéticos”, que abrange o sexo masculino e população negra. Todavia, ele foi mantido, por existir estudos que demonstram que esse tipo de sexo apresenta maior probabilidade de atraso do desenvolvimento^(13)^. Além disso, a população negra também deve ser colocada como um fator que está diretamente relacionado ao atraso no desenvolvimento, devido aos desafios significativos enfrentados por essa população, como racismo, desigualdades sociais e acesso limitado aos serviços essenciais^(14,15)^.

A seguir, está apresentado no Quadro 1 todos os elementos estruturais do diagnóstico de enfermagem Desenvolvimento Atrasado do Lactente, após a fase de análise de conteúdo pelos enfermeiros juízes.

**Quadro 1:** Elementos estruturais do diagnóstico de enfermagem Desenvolvimento Atrasado do Lactente, após a fase de análise de conteúdo pelos enfermeiros juízes – Redenção, CE, 2025.

Título
Desenvolvimento Atrasado do lactente
**Definição**
Lactente apresenta ausência de dois ou mais marcos nas áreas do desenvolvimento, caracterizadas por alterações cognitivas, comunicação/linguagem e pessoal/social.
**Fatores relacionados**
– Dificuldades na alimentação – Alterações no padrão de sono – Conhecimento inadequado dos pais/cuidadores sobre o Desenvolvimento do Lactente – Utilização de fórmula na criança menor de 6 Meses
**Características definidoras**
– Capacidade cognitiva prejudicada na aquisição da aprendizagem – Capacidade cognitiva prejudicada na resolução de problema – Atraso na linguagem do lactente – Lactente expressa dificuldade na linguagem receptiva – Lactente expressa dificuldade na linguagem expressiva – Dificuldade na interação social
**Populações em risco**
– Fatores genéticos: sexo masculino; população negra; – Fatores sociais desfavorecidos: ocupação paternal desfavorecida; família, economicamente, desfavorecida; falta de acesso à educação infantil; pouca variedade de brinquedos; Isolamento social da mãe nos primeiros anos de vida da criança; condições ambientais (moradia, abastecimento de água, presença de fossas sépticas e esgotos); – Ambiente familiar desfavorável: estresse parental; baixo estímulo; baixa escolaridade materna e dos cuidadores; presença de 2 ou mais irmãos; família faltando membro, principalmente a mãe; parentes de 1° grau com atraso na linguagem; baixa interação pai-filho; – Desfechos perinatais desfavoráveis: prematuridade; baixo peso; baixa estatura; baixo e alto perímetro cefálico; – Desfechos obstétricos desfavoráveis: idade < 20 anos; mães solteiras; multiparidade; abuso de substâncias na gestação (álcool, tabaco e outras drogas); gravidez não desejada; lactentes expostos ao HIV e outras infecções sexualmente transmissíveis; exposição pré-natal a poluentes do ar - NO2 e benzeno; – Maus-tratos à criança (físico, exposição à violência e negligência).
**Condições associadas**
Desnutrição; procedimentos invasivos (parto cesárea e fórceps, criança submetida à cirurgia cardíaca, admissão em cuidados intensivos neonatais); exposição prolongada a medicamentos (dexametasona, hidrocortisona em prematuros, anestésicos, antipsicóticos atípicos no período pré-natal, oxigenioterapia); fatores pré-natais e pós-natais (trombose venosa da placenta, restrição do crescimento intrauterino, obesidade materna, diabetes gestacional e desnutrição materna, depressão pré- e pós-parto, ansiedade pós-parto, distúrbio psíquico materno, infecções pré-natais não-tratadas); distúrbios neonatais (Apgar de 5 minutos <3, displasia broncopulmonar, asfixia perinatal com encefalopatia isquêmica hipóxica, hemorragia peri - intraventricular, sequelas da prematuridade); distúrbios neurológicos (epilepsia infantil, paralisia cerebral, retardo mental); distúrbio genético (síndrome(Sd) de Down, Sd. de Myrhe, Sd. de Williams); distúrbios congênitos (doença cardíaca congênita, deficiência visual profunda congênita, má formação); infecções neonatais não-tratadas (otite; sepse neonatal; infecções neonatais: citomegalovírus, zika vírus, HIV, Sífilis e Rubéola); atraso no crescimento linear e ponderal; transtorno do déficit de atenção com hiperatividade; autismo; perda auditiva; trauma cefálico; distúrbios metabólicos; distúrbios nutricionais: deficiência de vitamina B12.

## DISCUSSÃO

O diagnóstico de Enfermagem proposto apresentou alta concordância quanto à pertinência dos seus componentes entre os juízes, sendo classificados como relevantes, obtendo um percentual de concordância superior a 85% em quase todos os itens, o que sustenta a validade de conteúdo do presente diagnóstico. Assim, esse resultado evidencia que esses elementos estão alinhados com a prática clínica e com os conhecimentos teóricos sobre o desenvolvimento infantil, em especial na fase de lactente.

Já nos “Fatores sociais”, também pertencente às Populações em risco, foram acrescentados como pertencente a eles, às condições ambientais, de moradia, água, abastecimento e presença de fossas sépticas e esgotos, pois se sabe que a ampliação do acesso ao saneamento básico é um fator determinante para a redução de danos à saúde da criança^(16)^. Dessa forma, podemos considerar que os lactentes que vivem em condições ambientais e de moradia desfavoráveis, com ausência de tratamento e abastecimento de água, e ausência de fossas sépticas, vivem em condições de risco para o atraso do desenvolvimento^(17)^. Dessa forma, sua inclusão, validadas pelos especialistas como pertinentes, reforça a aplicabilidade do diagnóstico em contextos diversos, especialmente em regiões com menor acesso a recursos diagnósticos mais complexos.

Nas Condições associadas, foi elencada uma lista ampla agrupadas com componentes pertencentes a eles, sendo identificadas como múltiplas situações em que o enfermeiro necessita de outros profissionais para intervir de modo a obter resultado favorável, ou seja, são as condições que não são independentemente modificáveis pelo enfermeiro, contudo, é necessário conhecê-las. No presente estudo, foram elencadas condições como distúrbios genéticos, neurológicos e metabólicos, as quais são importantes no comprometimento do neurodesenvolvimento infantil e que, apesar de não serem modificáveis diretamente pela Enfermagem, constituem alertas clínicos para o rastreamento precoce e sistematização do cuidado por esses profissionais^(4)^. Além disso, a literatura afirma que diagnósticos de enfermagem validados são ferramentas facilitadoras para o acompanhamento adequado dessas condições, permitindo que o enfermeiro possa atuar de forma coordenada e em parceria com outros profissionais^(18)^.

Os Fatores relacionados foram considerados relevantes e bem aceitos nas duas avaliações, apenas o componente “Maus tratos” foi redirecionado para as condições associadas, por se tratar de uma exposição a um determinado evento, portanto, não fazendo parte dos FR. Além de ter sido acrescentado um novo elemento, “Utilização de fórmula na criança menor de seis meses”, sua inclusão vai de encontro a um estudo de revisão bibliográfica, onde demonstrou em seus resultados, artigos que evidenciaram que bebês em aleitamento materno exclusivo até os seis meses de idade tiveram um desenvolvimento neuropsicomotor melhor do que as amamentadas até os quatro meses, ou mesmo as que não amamentaram no primeiro ano de vida^(19)^. Outros fatores também merecem destaque, como “Conhecimento inadequado dos cuidadores” e “alterações no padrão de sono”, pois são reconhecidos pela literatura como modificadores ambientais do desenvolvimento. A partir deles, reforça-se o papel do enfermeiro na realização de atividades de educação em saúde e apoio às práticas parentais responsivas, no intuito de intervir ou mesmo prevenir a existência do atraso no desenvolvimento^(20)^.

No que tange às Características Definidoras (CD), do diagnóstico “Desenvolvimento Atrasado do lactente”, trazem um conjunto de sinais que estarão presentes quando a criança apresentar atraso no desenvolvimento. Na presente proposta, foram definidas, ao final, sete CD referentes a diferentes domínios, como: cognição, linguagem e pessoal-social. Foi necessária, durante a análise de consenso, a reformulação terminológica, seguindo a padronização da NANDA-I, com intuito de facilitar a comunicação entre os profissionais e operacionalizar o diagnóstico em diferentes contextos assistenciais. Além disso, a existência de múltiplas CD aumenta a acurácia diagnóstica, de acordo com Fehring^(7)^, fortalecendo, assim, planos assistenciais mais direcionados.

Dessa forma, as CD, que trazem relação com cognição e linguagem (capacidade cognitiva prejudicada na aquisição da aprendizagem e resolução de problemas, Atraso na linguagem do lactente e Lactente, expressam dificuldades na linguagem receptiva e expressiva) estão alinhadas com a literatura internacional, pois esta recomenda a observação sistemática dos marcos do desenvolvimento referentes a esses domínios com a finalidade de detectar precocemente o seu atraso^(21)^. Além disso, é necessário se atentar para a vigilância também dos aspectos psicossociais, como no caso da CD “Dificuldade na interação social”, pois a partir de sua identificação, essa criança poderá enfrentar desafios referentes à compreensão, respostas sociais e comunicação eficaz, aspectos encontrados em crianças com transtornos do neurodesenvolvimento^(22)^.

Portanto, ao identificar essas CD, permitirá ao enfermeiro a possibilidade de desenvolver intervenções voltadas para a promoção do desenvolvimento dessas habilidades em parceria com uma equipe multiprofissional.

As limitações do presente estudo referem-se à análise realizada apenas com profissionais da área de saúde da criança e/ou taxonomias de Enfermagem, o que pode limitar a diversidade de perspectivas acerca do diagnóstico proposto. Por outro lado, aumenta a especificidade sobre o tema. Outra limitação é referente à ausência de aplicação prática em contextos clínicos reais, sendo necessária futuramente a sua validação com público-alvo, visando fortalecer a aplicabilidade do diagnóstico na assistência de Enfermagem.

## CONCLUSÃO

O diagnóstico de Enfermagem proposto “Desenvolvimento Atrasado do Lactente” apresentou validade de conteúdo satisfatória, com elevada concordância entre os juízes quanto aos seus componentes, incluindo título, definição, fatores relacionados, características definidoras, populações em risco e condições associadas. A reformulação da nomenclatura das características definidoras de acordo com a linguagem da NANDA-I atrelada a evidências científicas, permite que esse diagnóstico se configure como uma ferramenta aplicável, útil e sensível à prática clínica de enfermagem. Além de possibilitar a contribuição do reconhecimento precoce de atrasos no desenvolvimento em uma faixa etária sensível como a do lactente.

## Data Availability

Todo o conjunto de dados que dá suporte aos resultados deste estudo está disponível mediante solicitação ao autor correspondente.

# Translation: Retraso en el desarrollo del lactante: análisis del contenido de una propuesta de diagnóstico de enfermeira *

**DOI:** 10.1590/1980-220X-REEUSP-2025-0400es

**Authors:** Maria do Socorro Távora de Aquino, Samara Pereira Souza Mariano, Flávia Paula Magalhães Monteiro, Huana Carolina Cândido Morais, Vanessa Emille Carvalho de Sousa Freire, Maria Nataniele Queiroz de Lima, Quéren-Hapuque Lopes Sousa

**Affiliations:** 1 Universidade da Integração Internacional da Lusofonia Afro-Brasileira, Instituto Ciências da Saúde, Redenção, CE, Brasil.

**Keywords:** Diagnóstico de Enfermería, Discapacidades del Desarrollo, Estudios de Validación

## Abstract

**Objetivo:**

Analizar el contenido del diagnóstico de enfermería «Retraso en el desarrollo del lactante».

**Método:**

Estudio metodológico, abordando la fase de análisis de contenido, utilizando un enfoque mixto. La primera etapa fue un estudio de concordancia cuantitativo; la segunda, un estudio de consenso cualitativo. Las dos etapas se llevaron a cabo con expertos. Para el análisis, se utilizó la prueba binomial, con un valor p igual o superior a 0,05 y un punto de corte de 0,85. El nivel de significación adoptado fue del 5%.

**Resultados:**

El diagnóstico propuesto obtuvo una alta concordancia entre los jueces en cuanto a su estructura (título, definición, características definitorias, factores relacionados, poblaciones en riesgo y condiciones asociadas), con más del 85% de aprobación. Algunas características se reformularon para alinearlas con la NANDA-I, mejorando la claridad y la aplicabilidad clínica.

**Conclusión:**

La validez del contenido fue satisfactoria, mostrándose relevante, clara y aplicable a la práctica de la enfermería. Su adopción puede favorecer la detección temprana de retrasos en el desarrollo infantil y respaldar intervenciones multiprofesionales, especialmente en contextos de vulnerabilidad.

## INTRODUCCIÓN

El desarrollo infantil (DI) abarca el espectro neuropsicomotor y puede dividirse, de manera resumida, en ámbitos, entre los que destacan el cognitivo, el psicosocial y el físico, que están interrelacionados y se influyen mutuamente a lo largo de la vida del individuo^(1)^. De este modo, las alteraciones en estos ámbitos pueden conducir a un retraso en el desarrollo. Sin embargo, para que se detecten tales alteraciones, el niño debe someterse a un seguimiento continuo, con el fin de detectar problemas de manera temprana, o incluso hacer un seguimiento del retraso cuando este ya se haya instalado, mediante instrumentos estandarizados y validados^(2)^.

No obstante, la tarea de identificar y acompañar a los niños con retraso en el desarrollo neuropsicomotor se enfrenta a la complejidad de factores como la inexistencia de un sistema de vigilancia eficiente, la falta de uso de instrumentos estandarizados y adecuados para realizar el cribado o incluso el hecho de que las pruebas consideradas como el estándar de referencia en la evaluación del retraso del desarrollo no sean gratuitas^(3)^.

El período en el que estos factores influirán más fuertemente dependerá, también, de cada fase o etapa designada de acuerdo con el ciclo vital del niño y su edad. Según los grupos de edad considerados por el Ministerio de Salud (MS), es en la fase de lactancia, que abarca desde los 29 días hasta los 24 meses de edad, donde se producen los cambios más grandes y rápidos en el DI, debido a la mayor plasticidad cerebral. En consecuencia, los niños que se encuentran en esta fase también están expuestos a problemas, como retrasos en el DI. Sin embargo, también responden mejor a las terapias y los estímulos cuando se les diagnostica de manera temprana^(4)^.

El enfermero es uno de los profesionales que puede realizar la detección del DI mediante el uso de diagnósticos de enfermería con el fin de orientar sus intervenciones. No obstante, no existe un diagnóstico de enfermería que especifique cada grupo de edad y, como se mencionó anteriormente, hay un grupo de edad, en particular, más sensible al diagnóstico y la intervención para el retraso en el desarrollo: la etapa de la primera infancia. A partir de esto, un estudio anterior^(5)^ construyó los elementos estructurales de un diagnóstico de enfermería enfocado en el retraso en el desarrollo del lactante. Sin embargo, aún no se había llevado a cabo la etapa de análisis de contenido, que es fundamental para el proceso de validación final del diagnóstico.

Este análisis de contenido se refiere a una de las etapas para la construcción o validación de un diagnóstico de enfermería, cuyo objetivo es examinar críticamente sus elementos estructurales, como el título, la definición, los factores relacionados, las características definitorias, las poblaciones en riesgo y las condiciones asociadas, mediante criterios psicométricos y la participación de evaluadores expertos, que garanticen que estos componentes sean coherentes con la práctica clínica y con un lenguaje estandarizado de enfermería, como el de NANDA-Internacional^(6)^.

Cabe destacar, además, que el uso de diagnósticos de enfermería validados permite la elaboración de planes de atención más eficaces, lo que contribuye a la promoción de la salud infantil y a la prevención de afecciones. Sin embargo, para que esta herramienta se aplique de manera eficiente, es necesario que su contenido sea previamente analizado y evaluado por especialistas, garantizando su pertinencia y claridad en la práctica clínica.

Ante esto, el objetivo de este estudio fue analizar el contenido del diagnóstico de enfermería “Retraso en el desarrollo del lactante”, utilizando un enfoque mixto.

## MÉTODO

### Diseño del Estudio

Estudio metodológico que aborda la fase de análisis de contenido, utilizando un enfoque mixto con un diseño exploratorio secuencial. La primera etapa fue un estudio de concordancia, cuantitativo, y la segunda, un estudio de consenso, cualitativo. Ambas etapas se llevaron a cabo con expertos en el área. Sin embargo, en la segunda etapa se aplicó un mayor rigor en la selección de estos evaluadores, considerando principalmente a aquellos con doctorado y que trabajaran o investigaran en el área materno-infantil y/o en taxonomías de enfermería. Esto se debió a que ellos evaluarían tanto las discrepancias de la primera etapa como reevaluarían los ítems que consideraran pertinentes.

### Lugar y Período

El estudio se llevó a cabo de manera remota. La primera etapa se realizó entre noviembre de 2018 y enero de 2019, de manera asincrónica, mediante contacto por correo electrónico. La segunda etapa se llevó a cabo entre octubre de 2020 y enero de 2021, y se realizó tanto de manera asincrónica, también por correo electrónico, como sincrónica, a través de la plataforma *Google Meet*.

### Población, Muestra y Criterios de Selección

En ambas etapas, la selección de los evaluadores/expertos se realizó mediante una búsqueda en la Plataforma Lattes del Currículo Lattes de investigadores, disponible en el portal del Consejo Nacional de Desarrollo Científico y Tecnológico (CNPq), utilizando los siguientes términos: análisis de concepto; diagnósticos de enfermería; crecimiento y desarrollo infantil; salud infantil; análisis de contenido y validación de contenido; y mediante muestreo en red o bola de nieve (*snowball*), durante el cual se solicitó la indicación de otro investigador que pudiera cumplir con los criterios de elegibilidad de este estudio, y la invitación se realizó a través del correo electrónico de cada evaluador.

Con el fin de considerar a dicho evaluador apto para realizar la evaluación, se aplicaron los criterios de Fehring^(7)^, de forma adaptada, y se tuvieron en cuenta también las recomendaciones de Lopes et al.^(8)^, que mencionan el equilibrio de los participantes en cuanto a la experiencia clínica y el conocimiento teórico. Inmediatamente después de la selección, se invitó a los evaluadores y se los dividió por perfil: el primero, formado por enfermeros clínicos con al menos cinco años de experiencia trabajando en el área de salud infantil y desarrollo infantil; y el segundo, formado por evaluadores con experiencia en el diagnóstico de enfermería y la taxonomía de NANDA Internacional (NANDA-I).

De este modo, para el análisis de concordancia (1.ª etapa), el tamaño de la muestra se definió mediante un cálculo que considera la proporción esperada de sujetos en relación con una variable dicotómica y la diferencia máxima aceptable de dicha proporción. El cálculo se realiza mediante la fórmula: n = Zα2.P.(1-P)/d2, donde Zα se refiere al nivel de confianza en el intervalo, adoptado en 95%, lo que corresponde al valor tabulado de 1,96; P es la proporción de individuos que están de acuerdo con la pertinencia del componente del diagnóstico de enfermería, considerada en un 85%; y d es la diferencia de proporción considerada aceptable, que será del 15%, incluyendo un intervalo de 70 a 100%. De este modo, el cálculo final se determinó mediante n = 1,96 × 2,085 × 0,15/0,152, concluyéndose en un tamaño de muestra de 22 evaluadores^(8)^.

Para este momento, se reclutaron 135 enfermeros en el primer contacto. Se obtuvo la respuesta de 36 evaluadores, de los cuales cinco se negaron a participar en el estudio por motivos académicos y profesionales y nueve, aunque inicialmente aceptaron, no devolvieron la evaluación dentro del plazo establecido, por lo que fueron excluidos de la muestra. Así, la muestra final estuvo compuesta por 22 enfermeros evaluadores.

La segunda etapa del estudio se denominó análisis de consenso, ya que se buscó seguir los principios metodológicos característicos de este tipo de enfoque^(9)^. Al igual que en la definición de los criterios para la selección de los evaluadores, ya mencionados anteriormente, se adoptó además la estrategia de discusión en grupos con el objetivo de alcanzar un consenso entre los participantes sobre los ítems del diagnóstico. El proceso implicó rondas sucesivas de retroalimentación y discusión colectiva, en las que se tuvieron en cuenta los comentarios y sugerencias de los evaluadores para la revisión y el perfeccionamiento de los ítems propuestos, caracterizando así una etapa de validación por consenso entre especialistas.

El muestreo fue no probabilístico, a través del grupo focal (GF). En este tipo de enfoque, se determina un número de evaluadores de entre seis y 15. Se reclutó a 61 enfermeros en el primer contacto y se obtuvo la respuesta de 15 evaluadores, de los cuales ocho se negaron a participar en el estudio por motivos académicos y profesionales. La muestra final estuvo compuesta por siete enfermeros evaluadores.

### Recolección de Datos

Tras aceptar participar en el estudio, cada evaluador recibió, por correo electrónico, el Formulario de Consentimiento Libre e Informado y el cuestionario de evaluación elaborado en la fase de análisis conceptual, con los elementos del diagnóstico propuesto “Retraso en el desarrollo del lactante”, que contenía título, definición, características definitorias, factores relacionados, poblaciones en riesgo y condiciones asociadas. Para establecer el consenso entre los evaluadores, con el fin de orientar los indicadores que se analizarían en el instrumento, se utilizó la relevancia como uno de los criterios psicométricos propuestos por Pasquali (1999)^(10)^, estableciéndose una escala tipo Likert con 5 opciones de respuesta, en la que los ítems 1, 2 o 3 se clasifican como inadecuados/irrelevantes; mientras que los ítems 4 y 5 se consideran adecuados/relevantes.

Con el fin de establecer un consenso colectivo entre los evaluadores, se realizaron reuniones a través de la plataforma *Google Meet*, en las que se estableció la metodología del GF. Se llevaron a cabo dos encuentros, con una duración de dos horas cada uno. El grupo fue conducido por un mediador, uno de los investigadores del estudio, y ambos encuentros fueron grabados para su posterior registro y análisis. Estos mismos evaluadores ya habían evaluado el instrumento previamente de manera individual, y los datos de la evaluación previa fueron presentados al GF por el mediador y discutidos por ambos. Todas las preguntas y sugerencias se discutieron hasta alcanzar el consenso, el cual se consideró alcanzado cuando la mayoría del grupo estaba de acuerdo con la síntesis de la discusión sobre cada pregunta y sugerencia realizada por el mediador.

### Análisis y Tratamiento de los Datos

Los datos se recopilaron en el programa Excel® 2016 y, posteriormente, para la tabulación, el cálculo de las medias y las pruebas estadísticas, se utilizó el programa *Statistical Package for the Social Sciences* (SPSS), versión 25.0.

El análisis estadístico de concordancia, según cada ítem del instrumento, se realizó mediante el ajuste de las proporciones de los evaluadores que estuvieron de acuerdo con la pertinencia del instrumento. Para ello, se empleó la prueba binomial, siendo pertinente el valor p igual o superior a 0,05 con un punto de corte de 0,85. Para este análisis, el nivel de significación (α) adoptado fue del 5%.

### Aspectos Éticos

Se cumplieron los preceptos de la Resolución 466/2012 del Consejo Nacional de Salud que trata de la investigación con seres humanos. El proyecto se registró en la Plataforma Brasil y fue aprobado por el CEP/UNILAB, protocolo n.º 5.032.639.

## RESULTADOS

Para el estudio de concordancia, los enfermeros presentaron una tasa de respuesta de los instrumentos del 16,2%. De ellos, el 95,5% (n = 21) eran mujeres, el 59,1% (n = 13) trabajaban en instituciones de educación superior, el 63,3% (n = 14) tenían un título de doctorado y el 36,4% (n = 8) tenían una especialización en el área de la salud infantil.

En cuanto a la actividad en la línea de investigación en salud infantil, el 77,3% (n = 17) mencionó que ya había trabajado o trabajaba en ella, y el 45,5% (n = 10) presentaba experiencia en el área del diagnóstico de enfermería.

Las variables edad y tiempo de formación presentaron una distribución asimétrica (p < 0,05), lo que indica que la mediana de edad fue de 33,50 (±8,78) años, siendo la edad mínima de 27 años y la máxima de 59 años. En cuanto al tiempo de formación, la mediana fue de 10 (±7,21) años, siendo el tiempo mínimo de cinco años y el máximo de 35 años.

En el estudio de consenso, todas las participantes eran mujeres. En cuanto a la titulación, dos (28,6%) enfermeras tenían especialización en el área de desarrollo infantil, una en neonatología y todas (100%) eran doctoras. Además, el tiempo de formación varió entre 10 y 20 años. Todas trabajaban como docentes en universidades de diversos estados, como Ceará, Paraíba, Bahía, Piauí, Goiás y São Paulo, y se dedicaban a temas relacionados con la salud infantil y/o taxonomías de enfermería.

Los resultados sobre la evaluación del contenido y el consenso de los elementos del diagnóstico propuesto se organizarán en tablas según los siguientes temas: evaluación del título y la definición, evaluación de la población en riesgo y condiciones asociadas, evaluación de las características definitorias y evaluación de los factores asociados.

### Evaluación del Título y la Definición

Para el análisis de contenido, se envió una opción de título del diagnóstico y tres definiciones elaboradas previamente durante el análisis conceptual, así como opciones de justificación y sugerencias, en caso de que se consideraran necesarias. Cabe destacar que, en la etapa de análisis conceptual, se realizó una lectura minuciosa de los artículos seleccionados para la elaboración de las definiciones.

En la Tabla 1 se encuentra la valoración sobre la idoneidad del título del diagnóstico de enfermería propuesto y su respectiva definición final elegida por los evaluadores.

**Tabla 1:** Evaluación de la adecuación del título y la definición para el diagnóstico de enfermería propuesto – Redenção, CE, Brasil, 2022.

Elementos	n	%
**Título:** Retraso en el desarrollo del lactante.	22	100
**Definición:** ”Retraso significativo en dos o más áreas del desarrollo, caracterizado por alteraciones que pueden incluir el rendimiento motor (grueso o fino), la comunicación/el lenguaje, el ámbito cognitivo, el ámbito social y las actividades de la vida diaria. El retraso se define como alteraciones ≥ 2 desviaciones estándar por debajo de la media”.	20	90,9

Se puede observar que hubo una gran concordancia en los dos elementos presentados. No obstante, los expertos del análisis de consenso sugirieron algunas modificaciones en la redacción para que fuera mejor aceptada por la taxonomía NANDA-I durante su presentación. De este modo, el título pasó a ser “Retraso en el desarrollo del lactante” y la definición: “El lactante presenta la ausencia de dos o más hitos en las áreas del desarrollo, caracterizados por alteraciones cognitivas, de comunicación/lenguaje y personales/sociales”, lo cual se justifica por ser más completo al incluir los ámbitos en los que puede producirse el retraso. Cabe destacar que todas estas modificaciones fueron aceptadas, ya que se trataba de profesionales con amplia experiencia en esta área y que estaban evaluando en conjunto, existiendo consenso entre todos respecto a estas modificaciones.

### Evaluación de la Adecuación de las Poblaciones en Riesgo y las Condiciones Asociadas

A continuación, en la Tabla 2 se presentan los indicadores que compusieron las poblaciones en riesgo y las condiciones asociadas mediante el análisis de conceptos, según el juicio basado en el índice de concordancia de los evaluadores en cuanto al criterio de relevancia.

**Tabla 2:** Índice de concordancia de los evaluadores respecto a la relevancia de los indicadores de las poblaciones en riesgo y las condiciones asociadas – Redenção, CE, Brasil, 2022.

Poblaciones en riesgo	n	%	p^*^
Factores sociales (Ocupación paterna desfavorecida; Familia en situación económica desfavorable; Falta de acceso a la educación infantil; Niños que viven en países en desarrollo; escasa variedad de juguetes; Aislamiento social de la madre durante los primeros años de vida del niño)	22	100	0,028
Entorno familiar desfavorable (Estrés parental; Bajo nivel de estimulación; Bajo nivel educativo de la madre; Cuidadores sin educación superior; Presencia de dos o más hermanos; Falta de un miembro de la familia, principalmente la madre; Parientes de primer grado con retraso en el lenguaje; Baja interacción padre-hijo)	22	100	0,028
Condiciones neonatales (prematuridad; bajo peso; baja estatura; bajo perímetro cefálico)	22	100	0,028
Condiciones obstétricas (edad < 20 años; madres solteras; multiparidad; abuso de sustancias durante el embarazo (alcohol, tabaco y otras drogas); embarazo no deseado; lactantes expuestos al VIH; Exposición prenatal a contaminantes atmosféricos: NO_2_ y benceno)	21	95	0,137
Factores genéticos (Sexo masculino; Origen étnico no blanco)	13	59	0,003
**Condiciones asociadas**	**n**	**%**	**p** ^*^
Exposición a medicamentos (uso neonatal de dexametasona; tratamiento prolongado con hidrocortisona en lactantes prematuros; exposición prolongada a anestésicos; exposición a antipsicóticos atípicos durante el período prenatal)	22	100	0,028
Trastornos neonatales (Índice de Apgar a los 5 minutos <3; Displasia broncopulmonar; Asfixia perinatal con encefalopatía isquémica hipóxica; Hemorragia peri-intraventricular; Secuelas de la prematuridad)	22	100	0,028
Trastornos neurológicos (Epilepsia infantil; Parálisis cerebral; Retraso mental)	22	100	0,028
Trastornos genéticos (síndrome de Down; síndrome de Myrhe; síndrome de Williams)	22	100	0,028
Trastornos congénitos (cardiopatía congénita; discapacidad visual profunda congénita)	22	100	0,028
Traumatismo craneoencefálico	22	100	0,028
Trastornos metabólicos	22	100	0,028
Malformaciones (cerebrales; craneosinostosis; labio leporino)	22	100	0,028
Trastorno por déficit de atención con hiperactividad (TDAH)	21	95	0,137
Autismo	21	95	0,137
Pérdida auditiva	21	95	0,137
Factores prenatales (Trombosis venosa placentaria; Restricción del crecimiento intrauterino; Obesidad materna; Diabetes gestacional)	21	95	0,137
Desnutrición infantil	20	91	0,338
Infecciones (Lactantes infectados por citomegalovirus; Otitis; Sepsis neonatal; Infecciones neonatales)	18	82	0,425
Procedimientos (Parto por cesárea; Niño sometido a cirugía cardíaca; Ingreso en cuidados intensivos neonatales; Oxigenoterapia)	17	77	0,226
Retraso en el crecimiento	17	77	0,028
Deficiencia de vitamina B12	17	77	0,226
Trastornos alimentarios	17	77	0,226
Trastornos maternos (Depresión prenatal y posparto; Ansiedad posparto; Trastorno psíquico materno)	16	73	0,000

^*Test binomial.^

Teniendo en cuenta que la hipótesis nula se representa mediante valores de concordancia entre los evaluadores iguales al 85%, el indicador de las poblaciones en riesgo relacionadas con las condiciones genéticas es significativo en lo que respecta a la falta de concordancia entre los evaluadores y, por lo tanto, debe eliminarse. Aún sobre este indicador, un juez sugirió separar sus componentes (sexo masculino y etnia no blanca) en grupos diferentes, sin asociarlos con condiciones genéticas.

Los demás indicadores tuvieron un índice de concordancia superior al 85% entre los evaluadores y un valor de p > 0,05 en la prueba binomial, por lo que fueron aprobados por los evaluadores. Sin embargo, hubo algunas sugerencias, como: en los “Factores sociales” se sugirió añadirles los elementos, condiciones ambientales, vivienda, agua, abastecimiento y presencia de fosas sépticas y alcantarillado. En “condiciones neonatales”, se sugirió agregar un perímetro cefálico elevado. En las condiciones obstétricas, se insertó el término Infecciones de Transmisión Sexual (ITS), además de la exposición al virus del VIH en el lactante. Ya en el análisis de consenso, los expertos mantuvieron las respuestas. Destacaron, además, la importancia de que cada elemento tenga sus componentes asociados. Además de la modificación del término “etnia no blanca” por “población negra” dentro de los factores genéticos. No se acordó, por lo tanto, su desglose, como se había sugerido anteriormente.

En lo que respecta a las condiciones asociadas, el indicador *desnutrición infantil* obtuvo una concordancia entre los evaluadores superior al 85%, sugiriéndose la eliminación del término “infantil”. Además, los indicadores *procedimientos, retraso en el crecimiento, malformación, deficiencia de vitamina B12, trastornos alimentarios e infecciones* obtuvieron un índice de concordancia inferior al 85%. Sin embargo, para la prueba binomial, estos deben mantenerse.

Por su parte, el indicador *trastornos maternos* obtuvo una concordancia inferior al 85% entre los evaluadores y un valor de p < 0,05 en la prueba binomial y, por lo tanto, debe retirarse. Un juez sugirió su retirada debido a que este indicador no se considera sólido en cuanto a su relación con el retraso en el desarrollo. En cuanto al indicador *trastornos alimentarios*, obtuvo un índice de concordancia inferior al 85% y un valor de p > 0,05, lo que indica que no hay significación estadística y que debe mantenerse. Un evaluador sugirió que este indicador debe revisarse junto con el indicador de desnutrición, ya que este último se inserta dentro de los trastornos alimentarios. El indicador *infecciones* obtuvo un índice de concordancia inferior al 85% y un valor de p > 0,05, lo que indica que no hay significación estadística y que debe mantenerse. Se sugirió su reformulación para incluir las infecciones no tratadas. También se sugirió añadir como factores de este indicador: el virus del Zika, el VIH, la sífilis y la rubéola.

Ya durante el análisis de consenso, se mantuvieron la mayoría de los elementos y se modificó únicamente la “malformación”, para incluirla dentro de los trastornos congénitos; la “deficiencia de vitamina B12” debería insertarse en los “trastornos alimentarios” y modificarse la nomenclatura a “trastornos nutricionales”.

### Evaluación de los Factores Relacionados y las Características Definidoras

En la Tabla 3 se presentan los factores relacionados (FR) y las características definitorias (CD), según el juicio basado en el índice de concordancia de los evaluadores.

**Tabla 3:** Índice de concordancia de los evaluadores en cuanto a los factores relacionados y las características definitorias – Redenção, CE, Brasil, 2022.

Factores relacionados	n	%	p
Dificultades para alimentarse	20	91	0,338
Maltrato	19	86	0,575
Alteraciones en los patrones de sueño	21	95	0,137
Conocimiento insuficiente de las madres sobre la DI	21	95	0,137
**Características definitorias**	**n**	**%**	**p**
Retraso cognitivo	22	100	0,028
Retraso en el aprendizaje	22	100	0,028
Retraso en la resolución de problemas	21	95	0,137
Retraso en el lenguaje	22	100	0,028
Retraso en el lenguaje receptivo	22	100	0,028
Retraso en el lenguaje expresivo	22	100	0,028
Ausencia de habilidades de comunicación verbal	21	95	0,137
Retraso psicosocial	22	100	0,028
Problemas socioemocionales	22	100	0,028
Problemas de socialización	16	73	0,100
Trastorno conductual	20	91	0,338
Conducta desafiante	20	91	0,338

En cuanto a los factores relacionados, se observa que todos los ítems presentaron una concordancia superior al 85% entre los evaluadores. Solo se sugirió que el elemento “Maltrato” se eliminara de los factores relacionados y se incluyera en las poblaciones en riesgo, lo cual fue aceptado. Ya durante el análisis de consenso, todos los expertos reafirmaron la importancia de mantener los mismos FR e insertar uno nuevo, titulado “Uso de fórmula en niños menores de seis meses”.

En cuanto a las características definitorias, a pesar de que todos los elementos obtuvieron una buena evaluación en la validación de concordancia, se sugirió la unificación de algunos por su similitud, como “Ausencia de habilidades en la comunicación verbal”, que se eliminó por estar ya contemplado en “Retraso en el lenguaje”; también se retiraron los elementos “Problemas socioemocionales” y “deficiencia conductual”, ya que pertenecían a otros elementos.

En la validación por consenso, se recomendó renombrar todas las características definitorias, con el objetivo de adaptarlas a la estructura y terminología utilizadas en los diagnósticos de enfermería, facilitando la comprensión y la aplicabilidad en la práctica clínica. Así, los términos “Retraso cognitivo” y “Retraso en el aprendizaje” se unificaron bajo la denominación “Capacidad cognitiva deteriorada en la adquisición del aprendizaje”. Del mismo modo, “Retraso cognitivo” y “Retraso en la resolución de problemas” se consolidaron en “Dificultad cognitiva en la resolución de problemas”. El término “retraso en el lenguaje” recibió la especificación de rango de edad, pasando a describirse como “retraso en el lenguaje del lactante”. Las expresiones “Retraso en el lenguaje receptivo” y “Retraso en el lenguaje expresivo” se reformularon como “El lactante expresa dificultad en el lenguaje receptivo” y “El lactante expresa dificultad en el lenguaje expresivo”, respectivamente. Además, los términos “Retraso psicosocial” y “Problemas de socialización” pasaron a denominarse “Dificultad en la interacción social” y, por último, “Comportamiento desafiante” pasó a llamarse “Presenta comportamiento socialmente inaceptable o indeseado”. Sin embargo, se sugirió la eliminación de este último, ya que no presentaba relación con los niños en el rango de edad de hasta 2 años. En un primer momento también obtuvo una puntuación baja. Por lo tanto, se aceptó.

A continuación, se presentan en el Cuadro 1 todos los elementos estructurales del diagnóstico de enfermería “Retraso en el desarrollo del lactante”, tras la fase de análisis de contenido por parte de los enfermeros evaluadores.

**Cuadro 1:** Elementos estructurales del diagnóstico de enfermería “Retraso en el desarrollo del lactante”, tras la fase de análisis de contenido por parte de los enfermeros evaluadores – Redenção, CE, 2025.

Título
Retraso en el desarrollo del lactante
**Definición**
El lactante presenta una falta de dos o más hitos en las áreas del desarrollo, caracterizada por alteraciones cognitivas, de comunicación/lenguaje y personales/sociales.
**Factores relacionados**
– Dificultades en la alimentación – Alteraciones en los patrones de sueño – Conocimiento insuficiente de los padres o cuidadores sobre el desarrollo del lactante – Uso de leche de fórmula en niños menores de 6 meses
**Características definitorias**
– Dificultades cognitivas en el aprendizaje – Dificultades cognitivas en la resolución de problemas – Retraso en el lenguaje del lactante – El lactante muestra dificultades en el lenguaje receptivo – El lactante muestra dificultades en el lenguaje expresivo – Dificultades en la interacción social
**Poblaciones en riesgo**
– Factores genéticos: sexo masculino; población negra; – Factores sociales desfavorables: ocupación paterna desfavorable; familia en situación económica desfavorable; falta de acceso a la educación infantil; escasa variedad de juguetes; aislamiento social de la madre durante los primeros años de vida del niño; condiciones ambientales (vivienda, suministro de agua, presencia de fosas sépticas y alcantarillado); – Entorno familiar desfavorable: estrés parental; escaso estímulo; bajo nivel educativo de la madre y de los cuidadores; presencia de dos o más hermanos; familia con un miembro ausente, principalmente la madre; parientes de primer grado con retraso en el lenguaje; escasa interacción padre-hijo; – Resultados perinatales desfavorables: prematuridad; bajo peso; baja estatura; perímetro cefálico bajo y alto; – Resultados obstétricos desfavorables: edad < 20 años; madres solteras; multiparidad; abuso de sustancias durante el embarazo (alcohol, tabaco y otras drogas); embarazo no deseado; lactantes expuestos al VIH y otras infecciones de transmisión sexual; exposición prenatal a contaminantes del aire: NO_2_ y benceno; – Maltrato infantil (físico, exposición a la violencia y negligencia).
**Condiciones asociadas**
Desnutrición; procedimientos invasivos (parto por cesárea y con fórceps, niños sometidos a cirugía cardíaca, ingreso en cuidados intensivos neonatales); exposición prolongada a medicamentos (dexametasona, hidrocortisona en bebés prematuros, anestésicos, antipsicóticos atípicos durante el período prenatal, oxigenoterapia); factores prenatales y posnatales (trombosis venosa de la placenta, restricción del crecimiento intrauterino, obesidad materna, diabetes gestacional y desnutrición materna, depresión pre y posparto, ansiedad posparto, trastorno psíquico materno, infecciones prenatales no tratadas); trastornos neonatales (puntuación de Apgar a los 5 minutos <3, displasia broncopulmonar, asfixia perinatal con encefalopatía isquémica hipóxica, hemorragia periintraventricular, secuelas de la prematuridad); trastornos neurológicos (epilepsia infantil, parálisis cerebral, retraso mental); trastornos genéticos (síndrome de Down, síndrome de Myrhe, síndrome de Williams); trastornos congénitos (cardiopatía congénita, discapacidad visual profunda congénita, malformaciones); infecciones neonatales no tratadas (otitis; sepsis neonatal; infecciones neonatales: citomegalovirus, virus del Zika, VIH, sífilis y rubéola); retraso en el crecimiento lineal y ponderal; trastorno por déficit de atención con hiperactividad; autismo; pérdida auditiva; traumatismo craneoencefálico; trastornos metabólicos; trastornos nutricionales: deficiencia de vitamina B12.

## DISCUSIÓN

El diagnóstico de enfermería propuesto mostró una alta concordancia entre los evaluadores en cuanto a la pertinencia de sus componentes, los cuales fueron clasificados como relevantes, obteniendo un porcentaje de concordancia superior al 85% en casi todos los ítems, lo que respalda la validez de contenido del presente diagnóstico. Así, este resultado evidencia que estos elementos están alineados con la práctica clínica y con los conocimientos teóricos sobre el desarrollo infantil, especialmente en la etapa de la primera infancia.

Los evaluadores o expertos eran, en su mayoría, mujeres y poseían un título de doctorado. Se observó que la tasa de respuesta más alta provino de los enfermeros que trabajaban en el área de salud infantil. Sin embargo, al buscar a estos profesionales, se procuró equilibrar a aquellos que poseían experiencia en el área de la salud infantil y en el área de las taxonomías de enfermería. De esta manera, la evaluación de los elementos del diagnóstico propuesto se vuelve más precisa, contextualizada y coherente, además de asegurar que estén alineados con la práctica asistencial y el lenguaje estandarizado de la enfermería^(11)^.

En cuanto al análisis de contenido, el título y la definición del diagnóstico propuesto fueron bien aceptados por los jueces en el análisis de concordancia. Sin embargo, durante el análisis de consenso, los expertos sugirieron modificar la forma de redactarlos, para que se asemejaran a la nomenclatura de la taxonomía de enfermería, especialmente la utilizada por NANDA-I, en la que se propondrá el diagnóstico. Esto se hizo con el fin de estandarizar dicha redacción, ya que, a partir de ello, podría contribuir a la comunicación eficaz entre los profesionales y a la continuidad de la atención^(12)^.

Las poblaciones en riesgo se refieren a grupos de personas que comparten características que hacen que cada una de ellas sea susceptible a una determinada respuesta humana. En el diagnóstico propuesto se enumeraron cinco grupos de poblaciones en riesgo con sus respectivos componentes. Uno de los que suscitó mayor desacuerdo entre los jueces fue el de los “Factores genéticos”, que abarca al sexo masculino y a la población negra. Sin embargo, se mantuvo, ya que existen estudios que demuestran que este tipo de sexo presenta una mayor probabilidad de retraso en el desarrollo^(13)^. Además, la población negra también debe considerarse como un factor directamente relacionado con el retraso en el desarrollo, debido a los importantes desafíos que enfrenta esta población, como el racismo, las desigualdades sociales y el acceso limitado a los servicios esenciales^(14,15)^.

En cuanto a los “Factores sociales”, que también pertenecen a las Poblaciones en riesgo, se han añadido las condiciones ambientales, de vivienda, agua, abastecimiento y la presencia de fosas sépticas y alcantarillado, ya que se sabe que la ampliación del acceso al saneamiento básico es un factor determinante para la reducción de los daños a la salud del niño^(16)^. De este modo, podemos considerar que los lactantes que viven en condiciones ambientales y de vivienda desfavorables, sin tratamiento ni suministro de agua, y sin fosas sépticas, viven en condiciones de riesgo de retraso en el desarrollo^(17)^. De este modo, su inclusión, validada por los especialistas como pertinente, refuerza la aplicabilidad del diagnóstico en diversos contextos, especialmente en regiones con menor acceso a recursos diagnósticos más complejos.

En las “Condiciones asociadas”, se enumeró una amplia lista agrupada con componentes que les pertenecen, identificándose como múltiples situaciones en las que el enfermero necesita a otros profesionales para intervenir a fin de obtener un resultado favorable; es decir, son las condiciones que el enfermero no puede modificar de manera independiente, sin embargo, es necesario conocerlas. En el presente estudio, se enumeraron condiciones como trastornos genéticos, neurológicos y metabólicos, los cuales son importantes en el deterioro del desarrollo neurológico infantil y que, a pesar de no ser modificables directamente por la enfermería, constituyen alertas clínicas para el rastreo temprano y la sistematización de la atención por parte de estos profesionales^(4)^. Además, la literatura afirma que los diagnósticos de enfermería validados son herramientas facilitadoras para el seguimiento adecuado de estas condiciones, lo que permite que el enfermero pueda actuar de manera coordinada y en colaboración con otros profesionales^(18)^.

Los factores relacionados se consideraron relevantes y bien aceptados en las dos evaluaciones; solo el componente “Maltrato” se redirigió a las afecciones asociadas, ya que se trata de una exposición a un evento determinado y, por lo tanto, no forma parte de los FR. Además de haberse añadido un nuevo elemento, “Uso de fórmula en el niño menor de seis meses”, su inclusión concuerda con un estudio de revisión bibliográfica, cuyos resultados mostraron artículos que evidenciaron que los bebés alimentados exclusivamente con leche materna hasta los seis meses de edad tuvieron un mejor desarrollo neuropsicomotor que los amamantados hasta los cuatro meses, o incluso que los que no fueron amamantados durante el primer año de vida^(19)^. Otros factores también merecen destaque, como el “conocimiento inadecuado de los cuidadores” y las “alteraciones en el patrón de sueño”, ya que son reconocidos en la literatura como modificadores ambientales del desarrollo. A partir de ellos, se refuerza el papel del enfermero en la realización de actividades de educación en salud y el apoyo a las prácticas parentales responsivas, con el fin de intervenir o incluso prevenir la existencia del retraso en el desarrollo^(20)^.

En cuanto a las Características Definitorias (CD) del diagnóstico “Retraso en el desarrollo del lactante”, estas aportan un conjunto de signos que estarán presentes cuando el niño presente un retraso en el desarrollo. En la presente propuesta, se definieron finalmente siete CD referidas a diferentes ámbitos, tales como: cognitivo, del lenguaje y personal-social. Durante el análisis de consenso, fue necesario reformular la terminología, siguiendo la estandarización de la NANDA-I, con el fin de facilitar la comunicación entre los profesionales y poner en práctica el diagnóstico en diferentes contextos asistenciales. Además, la existencia de múltiples CD aumenta la precisión diagnóstica, según Fehring^(7)^, fortaleciendo así planes de atención más específicos.

De esta manera, las CD relacionadas con la cognición y el lenguaje (capacidad cognitiva deteriorada en la adquisición del aprendizaje y la resolución de problemas; retraso en el lenguaje del lactante y del niño pequeño, que expresan dificultades en el lenguaje receptivo y expresivo), están en consonancia con la literatura internacional, ya que esta recomienda la observación sistemática de los hitos del desarrollo referidos a estos ámbitos con el fin de detectar precozmente su retraso^(21)^. Además, es necesario prestar atención también a la vigilancia de los aspectos psicosociales, como en el caso de la DC “Dificultad en la interacción social”, ya que a partir de su identificación, este niño podría enfrentar desafíos relacionados con la comprensión, las respuestas sociales y la comunicación eficaz, aspectos que se observan en niños con trastornos del desarrollo neurológico^(22)^. Por lo tanto, la identificación de estas DC permitirá al enfermero desarrollar intervenciones orientadas a promover el desarrollo de estas habilidades en colaboración con un equipo multiprofesional.

Las limitaciones del presente estudio se refieren al análisis realizado únicamente con profesionales del área de salud infantil y/o taxonomías de enfermería, lo que puede limitar la diversidad de perspectivas sobre el diagnóstico propuesto. Por otro lado, aumenta la especificidad sobre el tema. Otra limitación se refiere a la ausencia de aplicación práctica en contextos clínicos reales, siendo necesaria en el futuro su validación con el público objetivo, con el fin de fortalecer la aplicabilidad del diagnóstico en la atención de enfermería.

## CONCLUSIÓN

El diagnóstico de enfermería propuesto “Retraso en el desarrollo del lactante” presentó una validez de contenido satisfactoria, con una elevada concordancia entre los evaluadores en cuanto a sus componentes, incluyendo título, definición, factores relacionados, características definitorias, poblaciones en riesgo y condiciones asociadas. La reformulación de la nomenclatura de las características definitorias de acuerdo con el lenguaje de NANDA-I, vinculado a la evidencia científica, permite que este diagnóstico se configure como una herramienta aplicable, útil y sensible a la práctica clínica de enfermería. Además de permitir la contribución del reconocimiento temprano de retrasos en el desarrollo en un grupo de edad sensible como el del lactante.

## Data Availability

El conjunto de datos que respaldan las conclusiones de este estudio está disponible previa solicitud al autor correspondiente.
